# FRESH extrusion 3D printing of type-1 collagen hydrogels photocrosslinked using ruthenium

**DOI:** 10.1371/journal.pone.0317350

**Published:** 2025-01-10

**Authors:** Richard C. Steiner, Jack T. Buchen, Evan R. Phillips, Christopher R. Fellin, Xiaoning Yuan, Shailly H. Jariwala

**Affiliations:** 1 The Henry M. Jackson Foundation for the Advancement of Military Medicine, Inc., Bethesda, Maryland, United States of America; 2 Department of Physical Medicine and Rehabilitation, The Center for Rehabilitation Sciences Research, Uniformed Services University of Health Sciences, Bethesda, Maryland, United States of America; 3 CytoSorbents Medical Inc., Princeton, New Jersey, United States of America; University of the Witwatersrand, SOUTH AFRICA

## Abstract

The extrusion bioprinting of collagen material has many applications relevant to tissue engineering and regenerative medicine. Freeform Reversible Embedding of Suspended Hydrogels (FRESH) technology is capable of 3D printing collagen material with the specifications and details needed for precise tissue guidance, a crucial requirement for effective tissue repair. While FRESH has shown repeated success and reliability for extrusion printing, the mechanical properties of completed collagen prints can be improved further by post-print crosslinking methodologies. Photoinitiator-based crosslinking methods are simple and have proven effective in strengthening protein-based materials. The ruthenium and sodium persulfate photoinitiator system (Ru(bpy)_3_/SPS) has been suggested as an effective crosslinking method for collagen materials. Herein, we describe the procedure our group has developed to combine extrusion-based 3D printing of type-1 collagen using FRESH technology with Ru(bpy)_3_/SPS photoinitiated crosslinking methods to improve the strength and stability of 3D printed collagen structures. Mechanical testing and cell biocompatibility assessments were performed to investigate the impact of Ru(bpy)_3_/SPS photoinitiated crosslinking and highlight the potential limitations of this method. These results demonstrate a significant improvement in the compressive strength of type-1 collagen samples as the Ru(bpy)_3_/SPS concentration increases. Additionally, type-1 collagen samples crosslinked with up to 1/10 mM Ru(bpy)_3_/SPS support PC12 cell viability over a period of 7 days. The primary limitations that were observed and described in detail in this protocol are: the FRESH slurry preparation, printing environment, extrusion printer hardware, and quality of the ruthenium reagent.

## 1. Introduction

With over 200,000 peripheral nerve repairs performed annually in the US, peripheral nerve injury (PNI) is a significant clinical problem plaguing both military and civilian populations and affecting up to 2.8% of trauma patients [1,2]. The most severe form of PNI, neurotmesis, involves complete transection of the peripheral nerve, resulting in muscle degradation and complete loss of sensory and motor function [3]. The gold standard of interposing autologous donor nerve grafts between the stumps of a transected peripheral nerve suffers from several drawbacks, including donor site morbidity, nerve site mismatch, formation of neuromas, and limited supply of autologous nerve tissue, particularly in the case of military ballistic trauma resulting in multiple limb amputations [4,5]. Similarly, allografts and xenografts are limited in availability and face a significant risk of immunological rejection [6]. Therefore, it’s necessary to develop innovative approaches that overcome the limitations of the current standard of care.

Nerve guidance conduits (NGCs), tubular constructs composed of synthetic/natural biomaterials, are one such alternative that serve as biomechanical support structures to assist and guide regenerating nerves. Currently, there are a number of FDA-approved hollow, single-lumen conduits including NeuroMatrix®, Neuroflex® (Collagen Matrix Inc.), and NeuraGen® (Integra Lifesciences Corp.) [7,8]. However, these simple conduit designs lack the architectural complexity of native nerves and are limited to nerve gaps that are no larger than 3 cm. Therefore, the development of a nerve guidance conduit matching the effectiveness of autologous nerve grafts is deemed crucial to the field of PNI.

To fill the gap in treatment, design strategies have involved the development of primary scaffolds from biopolymers and synthetic polymers, as well as secondary internal scaffolds using luminal fillers such as laminin and fibronectin [9]. Optimization of these nerve guides has included surface micropatterning [10,11] stem cell inclusion [12,13] and controlled release of neurotrophic factors [14,15]. These designs have been demonstrated to improve peripheral nerve regeneration and offer flexibility in design [16–18]. However, none of these modifications in isolation has resulted in adequate functional recovery comparable to autograft treatments.

The rapid advancement of 3D printing presents a promising manufacturing method to fabricate biomimetic NGCs. Utilizing a layer-by-layer process, this technology employs computer-aided designs (CAD) to generate complex, high-resolution objects [19]. The flexibility in material varieties, the high degree of reproducibility, and the ease of modulating design parameters lend this technology to produce versatile and anatomically accurate nerve conduits [20]. Furthermore, cells, biomaterials, and biologically relevant molecules can be 3D printed in a sub-application of the technology known as bioprinting, providing a compelling strategy towards the inclusion of functional elements such as nanoparticles and bio-factors [21]. Overall, 3D printing offers the potential to develop conduits that meet both the anatomical and function requirements of native nerves, while simultaneously overcoming limitations of current grafting techniques.

Natural polymers are attractive candidates for the fabrication of NGCs, as they possess inherent biocompatibility, minimal immunogenicity, and the capability of natural enzymatic degradation [22–24]. Collagen, mainly type I, is a major component of the extracellular matrix spanning peripheral nerves and exists as fibrils along with collagen type-III and V in the epineurium, perineurium, and endoneurium layers ensheathing the adult peripheral nerve tissue [25]. Due to its high extrudability, collagen is compatible with 3D printing processes, however, being a soft and malleable material, it must be solidified, supported, or strengthened during printing, and crosslinked after printing to maintain the accuracy of 3D structures [26].

One successful method of supporting malleable biomaterials, such as collagen, while printing is the Freeform Reversible Embedding of Suspended Hydrogels (FRESH) technique developed by Hinton et al. in 2015 [27]. FRESH involves printing a hydrogel within a second sacrificial material that is thermoreversible and biocompatible. The FRESH process allows for 3D extrusion bioprinting of complex, biologically relevant designs, which were previously difficult or impossible to print due to the high malleability of many unmodified natural materials. Advanced BioMatrix has commercialized this technique utilizing acidified collagen inks and gelatin slurry medium for extrusion printing based on the protocol established by Hinton et al. in 2015 [27]. They supply their own proprietary inks (LifeInk 240) and support medium (LifeSupport) that have been validated by their own quality control assessment and from the research institutions they provide for and have demonstrated its use in FRESH 3D printing [28–30].

While collagen is bioactive, biodegradable, and possesses optimum cellular adhesivity, control of its biomechanical properties is limited [31]. Current strategies to modify the mechanical properties of collagen include the use of chemical crosslinking agents such as glutaraldehyde [32] and genipin [33], and through photopolymerization of methacrylate groups with ultraviolet (UV) light using photoinitiators such as 1-[4-(2-hydroxyethoxy)-phenyl]-2-hydroxy-2-methyl-1-propane-1-one (Irgacure 2959) [34], riboflavin and rose bengal [35,36]. Fancy & Kodadek [37] proposed a method based on the ruthenium-tris(2,2′-bipyridyl) dichloride/sodium persulfate (Ru(bpy)_3_/SPS) photoinitiator system to form dityrosine bonds between proteins using visible light (400–450 nm). Since collagen is inherently rich in tyrosine, this method requires no prior modification of the protein. Furthermore, this method utilizes visible light rather than UV, which has been shown to influence chromosomal and genetic instability in cells [38]. The proposed mechanism suggests that these reagents, in the presence of visible light, form Ru(III) and a sulfate radical. These intermediates then form tyrosine radicals, which dimerize to create dityrosine crosslinks. The crosslink density of the material is based on the tyrosine content of the protein [37,39–41].

Our research introduces a modified FRESH bioprinting protocol that is particularly relevant to the printing of collagen Type-1. It incorporates the FRESH technique with a pneumatic extrusion 3D-printing platform for the fabrication of collagen-based nerve guidance conduits, crosslinked using a biocompatible, visible light photoinitiator system (Ru(bpy)_3_/SPS). This method enables the effective printing of high-resolution nerve guidance conduits using the collagen-based biomaterial, LifeInk^Ⓡ^ 240. The effect of Ru(bpy)_3_/SPS photoinitiated crosslinking was investigated via mechanical analysis and *in vitro* cell viability of rat pheochromocytoma (PC12) cells. The following sections will detail the methods developed, the mechanical and cellular viability assessments performed, and discuss the implications of our findings in the broader context of 3D bioprinting as an effective modality for regenerative medical applications.

## 2. Materials and methods

The protocol described in this peer-reviewed article is published on protocols.io, https://dx.doi.org/10.17504/protocols.io.x54v92r2ml3e/v1 and is included for printing as S1 File with this article.

All materials were purchased from Sigma Aldrich unless noted otherwise. Lifeink^Ⓡ^ 240 and LifeSupport^Ⓡ^ were purchased from Advanced Biomatrix Inc (Carlsbad, CA). The Allevi 3 pneumatic extrusion 3D bioprinter was purchased from Allevi, Inc (Philadelphia, PA). Pheochromocytoma (PC12, CRL-1721™) cells and RPMI-1640 media (30–2001™) were purchased from ATCC^Ⓡ^ (Manassas, VA). Invitrogen™ LIVE/DEAD™ Viability/Cytotoxicity Kit and GIBCO™ penicillin-streptomycin were purchased from Thermo Fisher Scientific (Waltham, MA).

### 2.1. Bioink formulation

Lifeink^Ⓡ^ 240, an acidic type I collagen bioink (35 mg/ml), was used for extrusion-based FRESH 3D bioprinting. This product was shipped in a sterile 10 mL syringe, stabilized in an acetic acid/saline buffer solution and stored at 4°C upon arrival. The desired volume (2–3 ml) of the collagen ink solution was transferred into an appropriate 5 ml syringe using a luer lock syringe coupler. To remove any air bubbles from the ink solution, the syringe was centrifuged at 2000 G for 1 minute at 4°C, followed by removal of the air bubbles using hemostats to squeeze the syringe while pushing the plunger, allowing the air to escape. The prepared bioink syringe was then stored at 4°C or loaded for 3D printing of the conduits.

### 2.2. FRESH printing of collagen conduits

**Fig 1:**
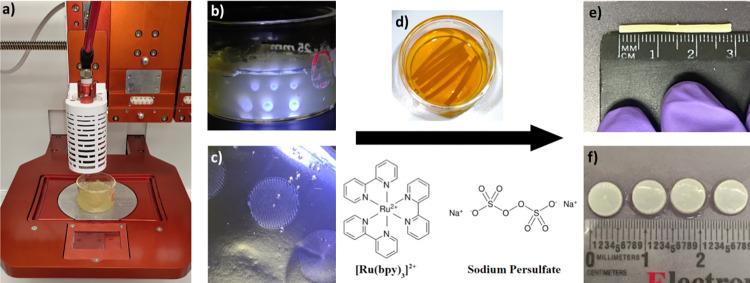
A representative schematic of the Lifeink^Ⓡ^ 240 printing process and crosslinking workflow including a) The Allevi 3 pneumatic extrusion printer, b-c) printed conduits and cylindrical samples of varying inner diameters, d) crosslinking solution of Ru(bpy)_3_/SPS, and e-f) crosslinked conduits and cylindrical samples.

3D bioprinting of Lifeink^Ⓡ^ 240 (35 mg/ml) was performed using the FRESH technique with version 2.0 LifeSupport^Ⓡ^ bath and the Allevi 3 pneumatic extrusion 3D bioprinter [27] (Fig 1A). LifeSupport^Ⓡ^ is a slurry of gelatin microparticles which was prepared as per Lee et al. [42]. Briefly, the desired amount of LifeSupport^Ⓡ^ powder was transferred into a 50 mL sterile conical centrifuge tube at 1 g of LifeSupport^Ⓡ^ powder per 35 ml of cold PBS and vortexed for 1 minute ensuring that all the powder is fully resuspended. The tube was allowed to sit for 24 hrs at 4°C to let the powder fully hydrate. The conical tube was then centrifuged at 2500 G for 5 minutes at 4°C. This modified Lifesupport (FRESH) protocol, the full protocol is included as supporting information file 1, reduces air bubbles and moisture and maximizes compaction of the gelatin slurry, thus providing consistent high-resolution collagen prints. The compacted LifeSupport^Ⓡ^ slurry was carefully transferred into a glass printing dish using a sterile spatula.

**Fig 2:**
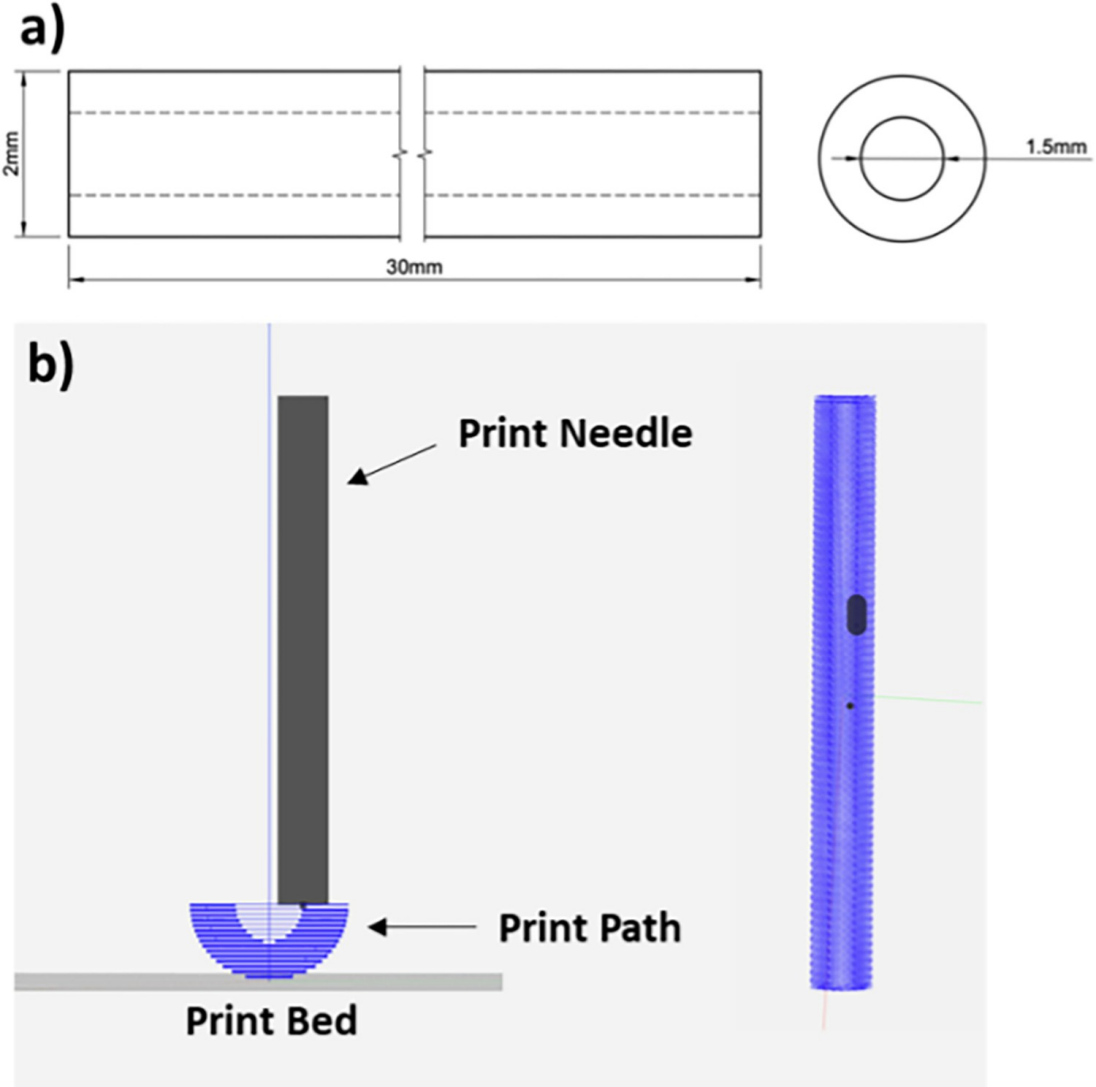
a) Schematic depicting a nerve guide conduit with the dimensions of 30 mm length, 2 mm outer diameter, and 1.5m m inner diameter. b) Schematic, displaying lateral and top views, of a sliced gcode file with its print path for a nerve guide conduit with the following parameters: 4 mm outer diameter, 1.5 mm inner diameter, 30 mm length, 50% infill, 150% overlap, no perimeters, 0.10 mm layer height, 0.17 mm extruder width.

All digital models of the conduits were created in computer-aided design (CAD) software (Fig 2A) Fusion (Autodesk), Shapr3D (Shapr3D Zrt), or TinkerCAD (Autodesk). Shapr3D was used for the final print design. All 3D models were then exported as an STL format file and processed in Repetier Host (Hot-World GmbH & Co. KG) and Slic3r slicing software to generate G-code instructions for the printer (Fig 2B). In general, slicer settings were set to a print speed of 6 mm/s, layer height of 0.10 mm, 0–2 perimeters, 150% perimeter overlap, 50% rectilinear infill, and 0.17 mm extrusion width. The collagen conduits were printed using an Allevi 3 pneumatic printer system (Fig 1A). Prints were obtained in a prepared life support bath medium supported in a glass petri dish using a 30-gauge needle (ID: 160 μm) with printing pressure of 50 psi at 4°C and at ambient temperature (21–23°C). Post-printing, conduits were incubated at 37°C for 45 minutes to allow thermal crosslinking of printed collagen Type-1 and melting of the LifeSupport™ slurry to release the bioprinted construct. Once released, the collagen constructs were washed with warm PBS (1X, pH 7.4, 37°C) solution to remove the melted gelatin support. Additionally, a group of collagen conduits and discs were photo-crosslinked for 15 minutes with Ru(bpy)_3_/SPS and visible light (1.65 mW, 425 nm) (Fig 1D). Briefly, 25, 50, or 75 mM Ru(bpy)_3_ and 250, 500, or 750 mM SPS stock solutions were prepared by dissolving both individually in 1X PBS. The washed 3D printed collagen conduits were placed in a petri dish containing PBS and solutions of Ru(bpy)_3_ and SPS were added at 2% (vol/vol) to achieve concentrations of 0.5/5, 1/10, and 1.5/15 mM Ru(bpy)_3_/SPS (Fig 1D). SPS was first added directly into the petri dish, followed by ruthenium. Conduits in the PBS-Ru(bpy)_3_/SPS bath were exposed to visible light (425 nm, 53.3 mW/cm^2^) placed 75 mm above the dish for 15 minutes to initiate crosslinking. After crosslinking, the conduits in the PBS-Ru(bpy)3/SPS solution were gently washed with deionized water (DI), and the conduits were stored in DI water at 4°C until used for specific biomechanical or in vitro testing. PBS-Ru(bpy)3/SPS solution and excess prep-reagent should not be stored and used for later use. Waste solution should be disposed of in chemical waste flasks using standard chemical waste disposal protocols.

### 2.3. Cell cytotoxicity live dead assay

In order to assess cell viability on the control and ruthenium crosslinked collagen conduit groups, ~7000 rat pheochromocytoma (PC12) cells were seeded on 3D printed collagen discs (n = 3) (6.28 mm diameter, 2 mm thickness) for each Ru(bpy)_3_/SPS concentration, and stained using an Invitrogen LIVE/DEAD™ Viability/Cytotoxicity Kit after 7 days in culture. PC12 cell cultures were maintained in RPMI-1640 media, supplemented with 10% horse serum, 5% fetal bovine serum, and 1% antibiotic solution penicillin-streptomycin under a humidified atmosphere with 5% carbon dioxide at 37°C. Tissue culture polystyrene served as a positive control for all cell culture studies. The full disk surface area was assessed qualitatively to determine cell health by two-photon confocal microscopy (Zeiss 7MP Multiphoton microscope). Folder with two-photon confocal microscope images are included as S2 File. Images were processed through ImageJ to filter areas of detected viable and non-viable cells to quantify disk surface viability by calculating the total percentage ratio of live to dead cells. Table showing percent ratios recorded from imageJ assessments are included as S2 Table.

### 2.4. Biomechanical compression testing

**Fig 3:**
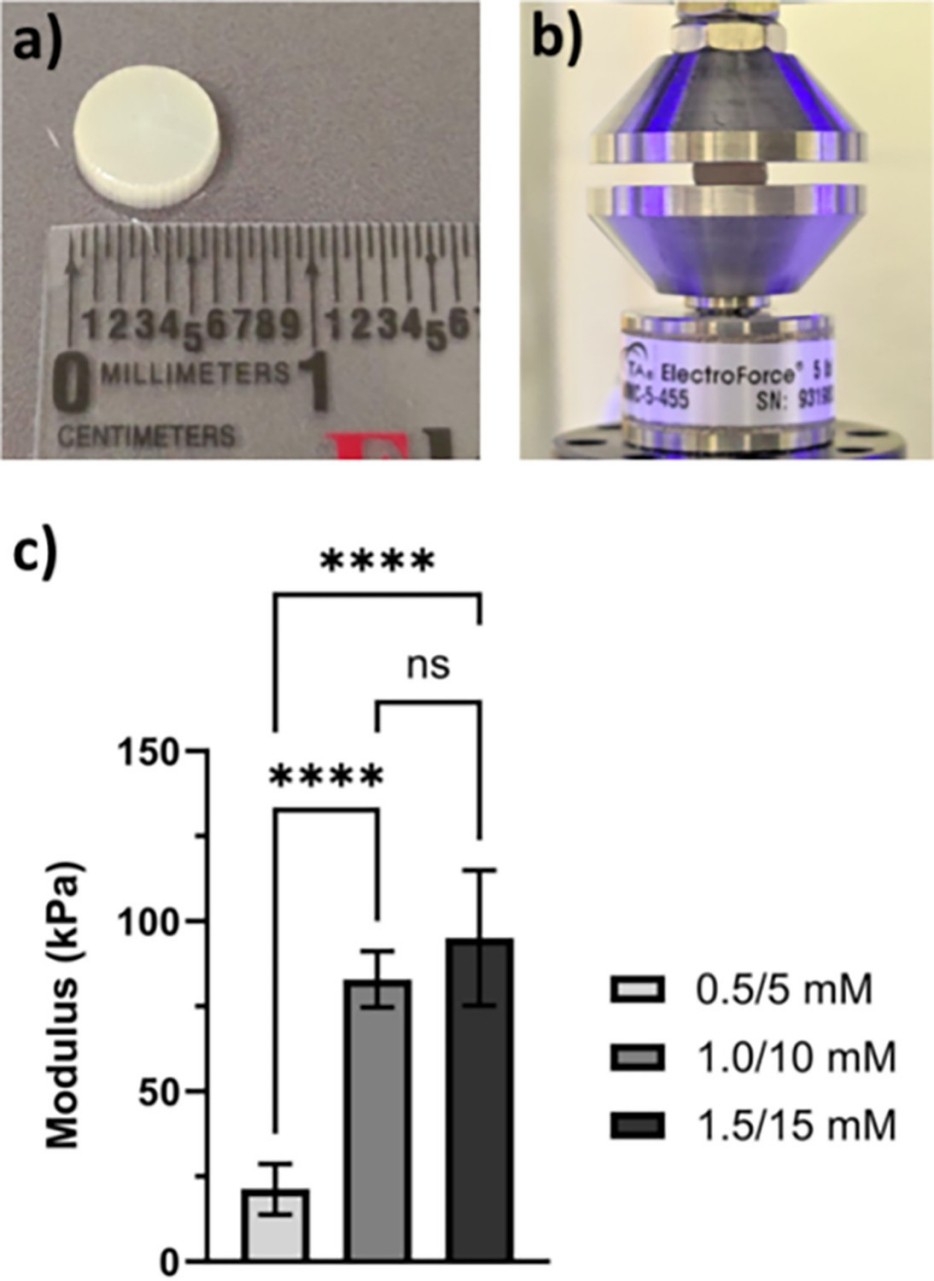
(a) Collagen disk measuring 6.28 mm x 2mm, used for compressive modulus testing (b) Electroforce DMA setup to assess compression modulus of Collagen Ru(bpy)_3_/SPS disks. (c) graphical representation of the compressive modulus of LifeInk 240 collagen disks at each crosslinker concentration (n = 6). ANOVA assessment (α = 0.05). Supplementary data of compression test provided in S1 Table.

3D printed collagen discs (n = 6) (6.28 ± 0.002 mm diameter x 2.0 ± 0.001 mm thickness) (Fig 3A) were fabricated and crosslinked using Ru(bpy)_3_/SPS (0.5/5 mM, 1/10 mM, and 1.5/15 mM) for unconfined compression testing (Fig 3B). The samples were allowed to equilibrate in 1X PBS for 24 hrs prior to testing on a dynamic mechanical analyzer (DMA, TA Electroforce 3220). Samples were loaded at a rate of 1 mm/min until failure was noticed or at 70% strain, whichever condition occurred first. The compressive modulus was calculated using the linear region of the recorded stress vs. strain curve prior to failure and averaged across the samples for a given crosslink group (Fig 3C). Table with compressive modulus calculations are included as S1 Table.

## 3. Expected results

This protocol further confirms and validates the ability of the FRESH technique to construct complex 3D designs using soft biomaterial inks by extrusion manufacturing. Depositing collagen bioink into a gelatin microsphere support medium gives collagen prints the structural reinforcement needed to create and hold complex 3D geometries. The stability of gelatin microspheres in a variety of temperature environments over long periods of time assists in maintaining the construct’s form without introducing construct collapse or ink path separation during the period required for printing and crosslinking of the collagen material.

The addition of the Ruthenium/Sodium Persulfate (Ru(bpy)_3_/SPS) photoinitiated crosslinking step has demonstrated a noticeable increase in the biomechanical properties of the collagen material while maintaining suitable biocompatibility towards neural model cell lines (PC12). Compression analysis showed a significant increase in compressive stress (21.88 ± 5.85 kPa to 87.89 ± 22.81 kPa) of 3D printed collagen samples as the concentration of Ru(bpy)_3_/SPS increases from 0.5/5 mM to 1.0/10 mM (Fig 3c). These results indicate successful tyrosine dimerization has occurred after visible light photoinitiation. However, there is no significant difference in compressive strength between samples crosslinked with 1.0/10 mM and 1.5/15 mM Ru(bpy)_3_/SPS, indicating that most available tyrosine moieties have already dimerized and additional photoinitiator (> 1.0/10 mM) does not result in more effective crosslinking.

**Fig 4:**
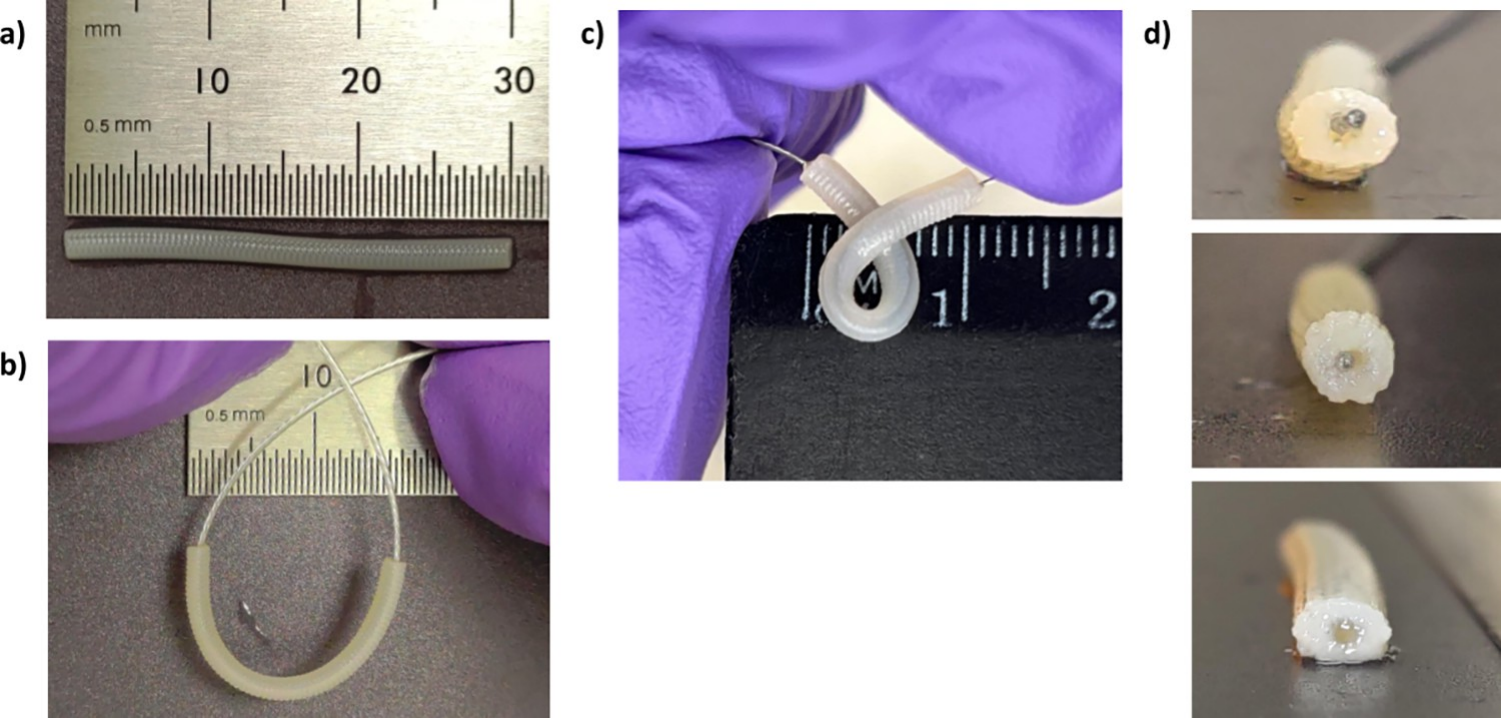
(a) 3cm long hollow, single lumen nerve guide conduit with 4mm outer diameter and 1.5mm inner diameter (b) overhead view of conduit bending without kink formation (c) overhead view of conduit bending with kink formation at the mid section of the conduit (d) intrusion of wire through the hollow section of the conduit.

The modified FRESH protocol, combined with Ru(bpy)_3_/SPS photoinitiated crosslinking, enabled the printing of hollow, single-lumen nerve guide conduits up to 5 cm in length (Fig 4A). These 5 cm conduits displayed excellent elasticity and strength, and offer promising applications in addressing severe neurotmesis. No limiting factors to the conduit’s structural integrity were observed at this length. This was determined by the absence of tears or delamination of print fibers or 3D structure by threading a thin wire through the hollowed sections and bending the conduit to create a kink at the mid section of the conduit (Fig 4B and 4C). This further demonstrates improvements in maintaining collagen 3D print shape and stability post organoruthenium crosslinking, thus an improvement in print fidelity and quality. Future research aimed at identifying the maximum effective length achievable with these bioprinting and bioink methods could enhance flexibility in addressing various severities of nerve injuries and potentially expand the range of applications currently constrained by the limitations of bioprinted collagen.

**Fig 5:**
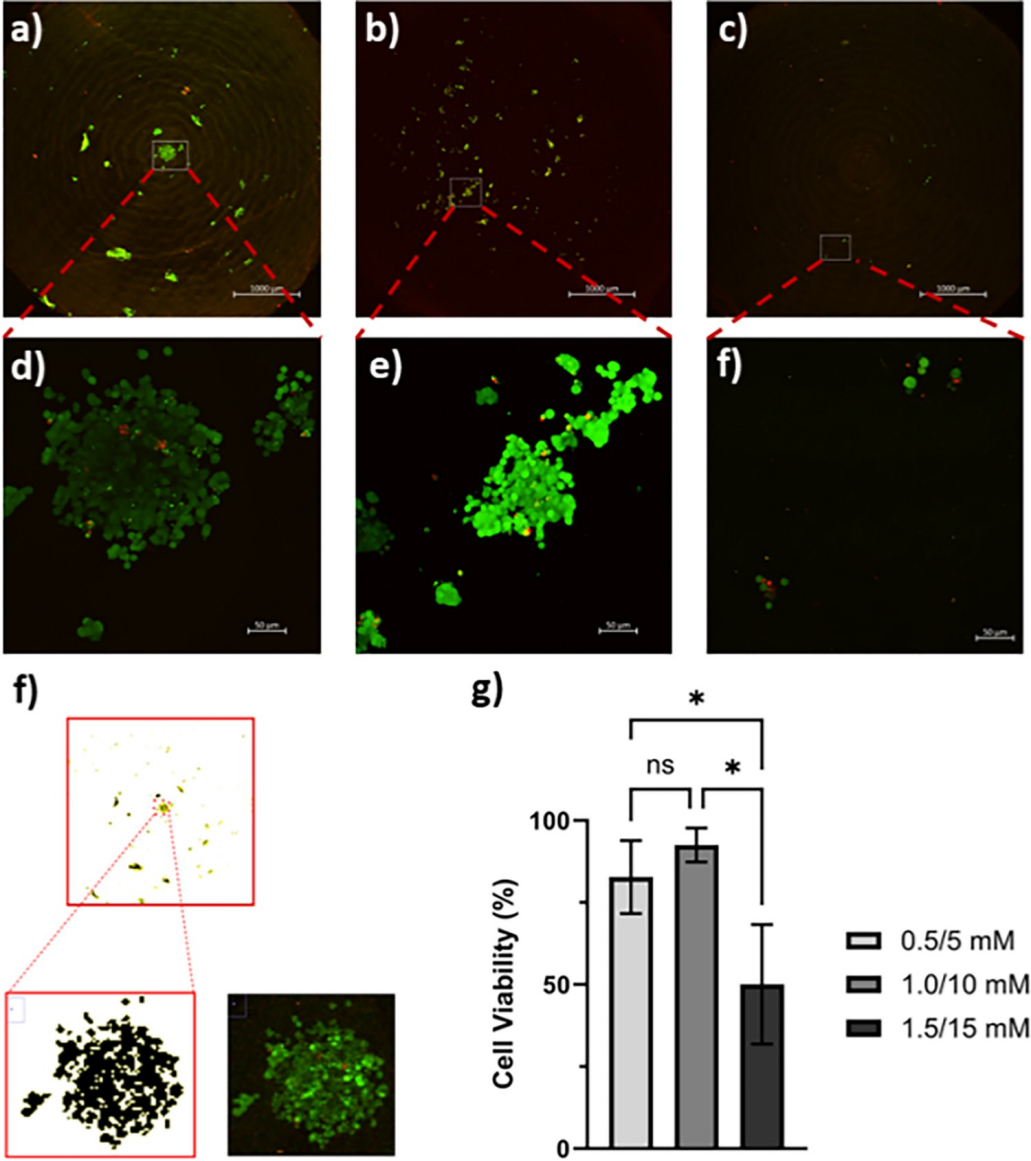
Live/Dead (Calcein AM = green and Ethidium Homodimer = red) staining of PC12 cell culture on the surface of LifeInk 240 collagen disks at Ru(bpy)_3_/SPS concentrations of (a, d) 0.5/5 mM, (b, e) 1.0/10 mM, and (c, f) 1.5/15 mM. f) Live-cell disk surface coverage assessment using image J software and g) Cell viability of PC12 cells after 7 days cultures on LifeInk 240 collagen disks for each Ru(bpy)_3_/SPS concentration (n = 3). Supplementary data of cytotoxicity assessment provided in S2 File and S2 Table.

PC12 cells cultured for 7 days on the surface of collagen disks showed stable cultures and high viability of 82.7 ± 11.1% and 92.5 ± 5.2% on disks crosslinked with 0.5/5 mM and 1.0/10 mM Ru(bpy)_3_/SPS, respectively. At concentrations greater than 1.0/10 mM, a significant drop in cell viability is observed (50.1 ± 18.2% at 1.5/15 mM) (Fig 5G). The residual Ru(bpy)_3_/SPS from higher concentrations (> 1.0/10 mM) may have a deleterious effect on cell viability. This hypothesis is supported by the findings of Gulzar et al. [41], who investigated the viability of 3T3-J2 murine fibroblast cells, human corneal epithelial cells, and human limbal mesenchymal stem cells after treatment of various concentrations of (0.1 μM—0.1 M) Ru(bpy)_3_/SPS and observed degraded viability with increased concentration across all samples [43].

## 4. Limitations

One of the most common limitations of collagen based hydrogels is their poor mechanical properties due to few covalent crosslinking potential among collagen fibers. This makes collagen hydrogels a very poor candidate for any application involving tissue load bearing or cyclical tension. The addition of Ru(bpy)₃/SPS crosslinking addresses this by adding tyrosine bonding anchors to the collagen fibers, thus increasing the amount of covalent bonding points between fibers. The mechanical compression results post-crosslinking show a significant improvement in load bearing resistance with a maximum strength around 80 kPa before failure, compared to 5% (w/v) GelMA crosslinked with Irgacure reaching only a maximum strength of 2.4 kPa [44]. There are a number of tissues whose tissue stiffness = <100 kPa would make Ru/SPS crosslinking of collagen based materials acceptable such as brain, muscle, tendon, peripheral nerve, or collagenous bone [45–47]. However, certain tissue types remain less effective of Ru(bpy)₃/SPS enhanced collagen hydrogels due to higher mechanical resistance requirements (>100 kPa) and issues pertaining to fluid dilution through hydrogel interfaces such as vascular and bone grafts. While studies have shown RU/SPS crosslinking of collagen materials is able to preserve cell viability and direct cells to differentiate towards osteogenic pathways, it must be supplemented with stronger load bearing materials as a hybrid hydrogel [48]. Most tissue grafts are mechanically validated by comparing the axial tension, compression, or bending strength to that of native tissue. For tissues with alternative force stressors, such as fluid flow, will need additional assessments such as burst pressure and shear stress.

PC12 is a tumor based cell line and therefore has different mechanisms and pathways compared to native neurons and ganglions. As a continuous cell line, it is capable of proliferating at a very high rate and is robust enough to survive a multitude of material environments. Other immortalized cell lines are often used to generally assess the biocompatibility of materials within in vitro conditions such as A549 or HeLa cells [49]. The differentiation of PC12 towards dendrite extension is also quite varied based on the biomaterial the cell interfaces with. Surface topography, material coating, and hydrophilicity can significantly impact PC12 differentiation/proliferation through mechanotransduction, chemotaxis, and cytotaxis mechanisms [50]. It is for this reason that PC12 can act as an acceptable cell line for most in vitro models and is one of the best candidates to test material biocompatibility within a neuron dense environment [51]. However, it is important to select an immortalized cell line based on its tissue origin that best matches the tissue application the material tested will be applied to.While this cell line is similar to sympathetic neurons and is often used to test biocompatibility neurodegenerative and neurotoxicity in neural tissue environments, its tumor origin makes cell morphology varied and unable to develop functional dendrites or axons with true synaptic formations [51,52]. This cell line is also limited by its nutritional and surface adhesion needs which usually require an environment high in serum albumin to grow and a surface that the cell can bind to which often utilizes surfaces covered with collagen, laminin, or poly-lysine. It is also restricted in its differentiation potential as they require Neurotrophic growth factor (NGF) in order to differentiate towards neural cell states. This makes PC12 suitable for in vitro conditions for material evaluation, but it will need to be further validated with other cell lines and in vivo models to thoroughly investigate its neurogenic potential such as with other neurogenic continuous cell lines or primary glial, ganglion, and neural cell lines.

Ru(bpy)₃/SPS cross linking has demonstrated to have excellent biocompatibility and minimal toxicity at ratios of between 0.5/5 to 1.0/10 mM (Ru/SPS) when used for strengthening collagen based materials [53]. A variety of cell types have been tested both in vitro and in vivo using this crosslinking method, including examples of corneal epithelial cells, fibroblasts, and our demonstration of PC12 cells [39,54]. An example study comparing the biocompatibility of using Ru/SPS vs Irgacure crosslinking for gelatin materials recorded greater cell cytocompatibility than Irgacure 2959 with cultures of HAC (human adrenocortical carcinoma) at up to 21 days [40]. Furthermore, it was found that high concentrations of Ru/SPS did not impede cell viability, unlike Irgacure which proved to be detrimental to cell viability at similar concentrations and at higher UV(ultra violet) light intensity.While most papers report a significant improvement in bulk material strength and stability, the primary limitations continue to be a small improvement in mechanical strength that is not capable in tissue repair applications involving high load bearing or tensile stressors. Studies have also demonstrated excellent viability and stability of stem cells seeded onto hydrogels crosslinked with Ru/SPS with no detectable impedance in the cells ability to differentiate towards its directed lineage [55,56]. As we have seen in many material validation tests, cell line performance can be impacted by mechanotransduction and chemotaxis pathways based on the stiffness and surface chemistry pattern of hydrogels. It is therefore possible that Ru/SPS can enhance or impede cell adhesion, health, and differentiation potential towards specific tissue lineages. This makes it important to consider the appropriate amount of crosslinker needed to tailor the hydrogel stiffness that best meets the tissue applications that the hydrogel is intended for. Further research will be needed to see how di-tyrosine bonding might further modify the surface or internal architecture of collagen hydrogels to bind certain protein/peptide motifs that can improve cell performance [57].

### 4.1. Slurry prep

While FRESH technique has consistently demonstrated its ability to produce complex collagen constructs, it is not without its limitations. In particular, the variability in print quality can be significant and is impacted by a number of variables including the gelatin slurry preparation, printing environment, quality of ruthenium photoinitiator, and type of extrusion printer used.

In order for the gelatin slurry to support the collagen bioink, it must have the necessary yield stress to not only hold the ink in a fixed position once it’s extruded, but it must also flow freely around the needle as it travels through the slurry. These types of slurry mediums are often characterized as Bingham plastics or Herschel–Bulkley fluids, and it is this trait that must be carefully considered when designing functional support slurries. If the slurry is not viscous enough the slurry will continue to flow after the ink is deposited, leaving the print with significant gaps and misalignments that negatively impact the structural integrity of the print. However, if the slurry is too viscous and dense the needle will have difficulty moving freely through the slurry and also cause misalignment and gaps in the print structure. Both issues were encountered during our initial printing attempts due to variability in the gelatin slurry mechanics, despite following the provider’s protocol for slurry preparation. Therefore, several procedures were designed to mitigate these issues. When the prepared gelatin slurries were not viscous enough, the temperature and humidity of the print environment was verified, and if necessary, adjusted to fall within the standard printing temperature of 17–22°C. If the desired temperature was lower than this range, ice packs were applied near or under the print stage. Ensuring a proper environmental temperature allowed for improved slurry viscosity, and therefore print quality. To further improve slurry viscosity and increase manufacturing efficiency, the number of centrifugation steps was altered and incorporated moisture reduction techniques. Centrifugation steps were reduced from 2 to 1, at a force of 2500 G. Low-linting wipes were used to remove excess PBS from the conical tube after centrifugation, which reduced excess PBS absorption by the slurry. These adjustments reduced the time needed for full hydration of the slurry from an hour to 10 minutes. Additionally, fixing the slurry dish to the print stage using adhesive tape was helpful in preventing the dish from sliding off axis due to the drag force created by the pathing of the extrusion needle through the slurry while printing.

### 4.2. Printing

As previously mentioned, there is a suggested working temperature for Lifeink^Ⓡ^ 240 bioink at around 4°C and a working temperature for the gelatin slurry during printing to be between 17–24°C. Variability in print outcomes can be further improved by incorporating an environmental control container to precisely regulate temperature and atmospheric conditions such as humidity.

### 4.3. Bioprinter selection

The type of extrusion printer used can impact extrusion parameters and thus print quality. Allevi 3 systems utilize pneumatic compressed air to propel the bioink through a syringe and needle. When the rubber syringe stopper does not create a stable seal, caused by improper syringe preparation or as a result of the high pressures used during printing, excess air can enter the ink reservoir. This can result in both a mixture of air and ink, as well as significantly lower pressure at the needle tip than what is produced by the air compressor. Complications from this process often include insufficient extrusion at the needle tip, as well as air bubbles trapped in the bioprint, which significantly reduces the resolution and structural integrity of the final print. Another issue common to extrusion printing applications that arose were air bubbles trapped in the luer lock connection of the needle/syringe interface. This is often very difficult to remove; however, manually extruding a small amount of bioink into the luer lock prior to connecting the needle to the syringe removes most of the air from the connection. Another process (gcode) was also implemented to rapidly extrude ink in short pulses prior to printing to clear any remaining air from the luer lock connection More information on the process of preparing the printer can be found in the protocol attached as supporting information file 1 with this article. Our group recommends the use of either piston or screw displacement extruders instead of pneumatic systems as they offer greater control over the consistency and reliability of the compressive force needed for extrusion, without the complication of introducing air into the system.

### 4.4. Ruthenium crosslinking

Lastly, we observed the quality of the ruthenium reagent changes over time. Ruthenium combined with sodium persulfate during light photoinitiation results in further crosslinking of the collagen print, but over time continued use of the ruthenium stock can result in a reduction in its functionality to create tyrosine bonds in the collagen structure. This was attributed to the exposure of ruthenium stock to peripheral light during periods of use, as well as excess moisture absorption caused by repeated use of the ruthenium stock. The ruthenium is stored in amber bottles during reagent preparation, but excess light or moisture from the room could cause degradation in ruthenium’s ability to create tyrosine bonds under light crosslinking over an extended period of time. In the future, all regent prep should be done with as minimal light as possible to prevent further chemical degradation. Moisture absorption by Ruthenium particulates can be addressed by storing the stock ruthenium bottle in a vacuum chamber until ready to use, then transfer into a chemical fume hood during reagent preparation. The prepared ruthenium reagent solution should be combined with sodium persulfate reagent in the collagen print solution immediately after it is prepared to ensure the crosslinking is at its most effective.

## 5. Conclusion

In conclusion, a detailed method for the extrusion-based 3D printing of type-1 collagen was developed using a modified FRESH technique and the biomechanical properties of printed structures were enhanced via photoinitiated crosslinking (Ru(bpy)_3_/SPS). The compressive strength of collagen samples increased after Ru(bpy)_3_/SPS photoinitiated crosslinking while maintaining high biocompatibility of PC12 cells at concentrations ≤ 1.0/10 mM up to day 7 in culture. Several limitations were also highlighted that have a significant impact on successful extrusion-based 3D printing of collagen materials using the FRESH protocol. These limitations should be considered and controlled for when repeating or altering this protocol.

## Supporting information

S1 File Protocol.io copy. (PDF)

S2 File PC12 Live/Dead. (ZIP)

S1 Table Compression data. (XLSX)

S2 Table PC12 Live/Dead. (XLSX)

S1 Protocol Final protocol document. (DOCX)
