# Correction: FRESH extrusion 3D printing of type-1 collagen hydrogels photocrosslinked using ruthenium

**DOI:** 10.1371/journal.pone.0326622

**Published:** 2025-06-17

**Authors:** The PLOS One Staff

In the Author Contributions, Xiaoning Yuan should be listed as one of the persons who contributed to Funding Acquisition.

The publisher apologizes for the error.
