# Supplementary figures and images for: FRESH extrusion 3D printing of type-1 collagen hydrogels photocrosslinked using ruthenium
Source: PLoS One. 2025 Jan 10;20(1):e0317350. doi: 10.1371/journal.pone.0317350 (PMC11723599; doi:10.1371/journal.pone.0317350)

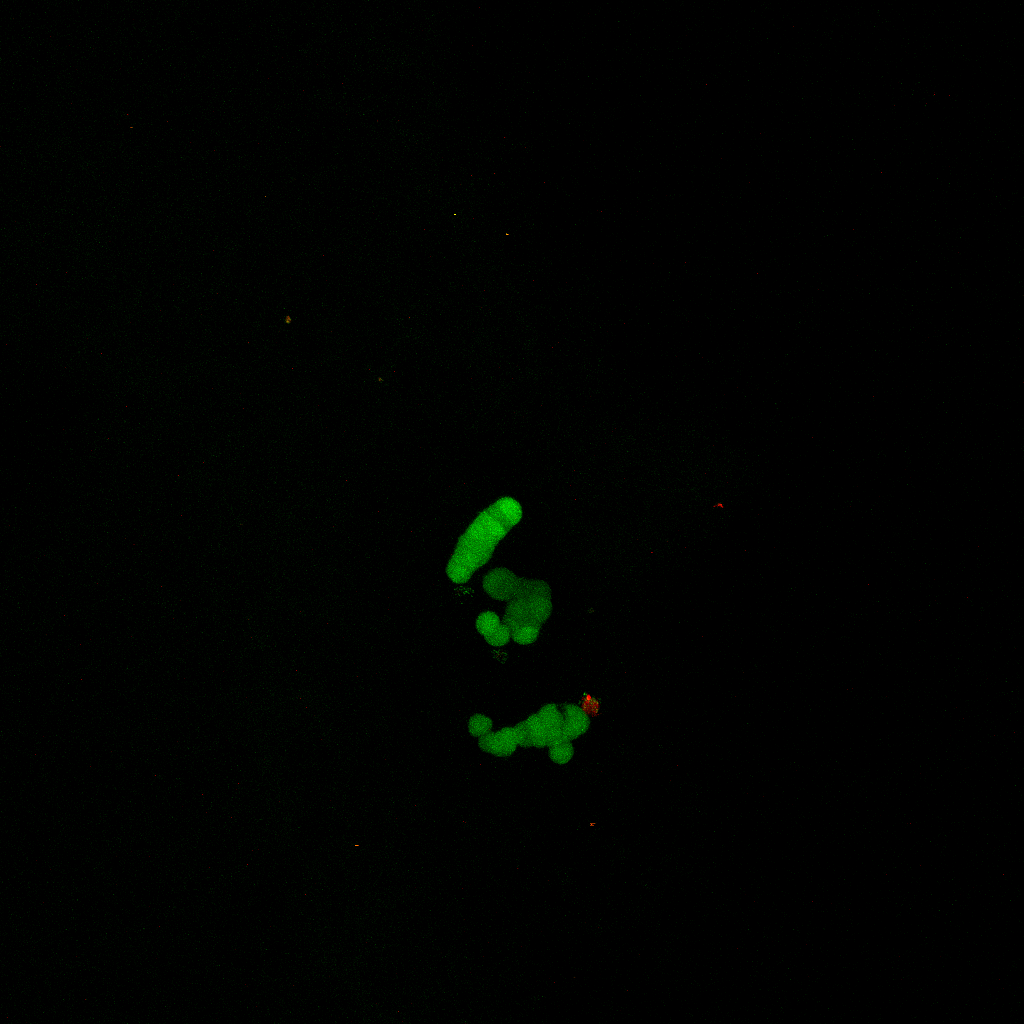

Supplement: S2 File — (ZIP) [file pone.0317350.s002.zip › S5_Image Folder/011922/011922_7 days_1.5-15mM RU-SPS_Disk 210707_20x_S1_Maximum intensity projection_b0v0t0z0c0-2x0-1024y0-1024.tif]

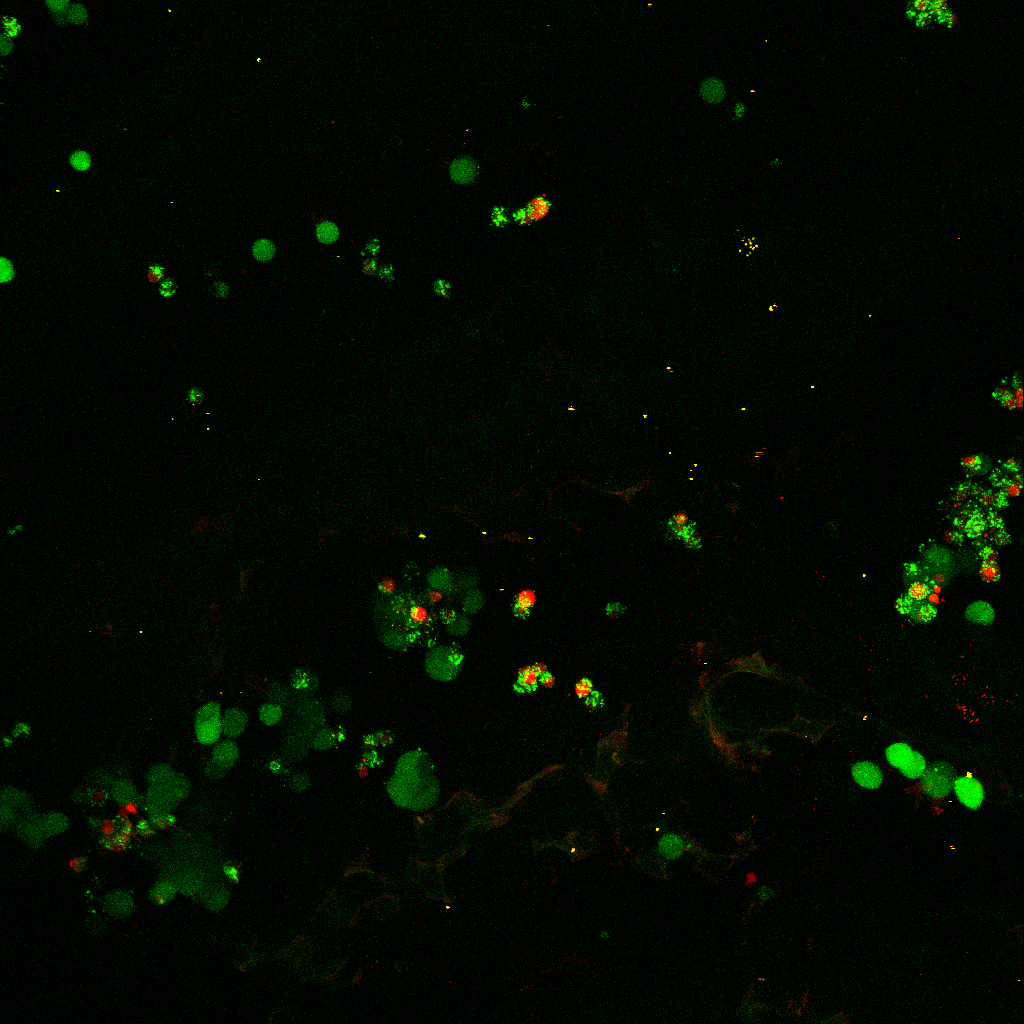

Supplement: S2 File — (ZIP) [file pone.0317350.s002.zip › S5_Image Folder/011922/011922_7 days_1.5-15mM RU-SPS_Disk 210707_20x_S2_Maximum intensity projection_b0v0t0z0c0-2x0-1024y0-1024.tif]

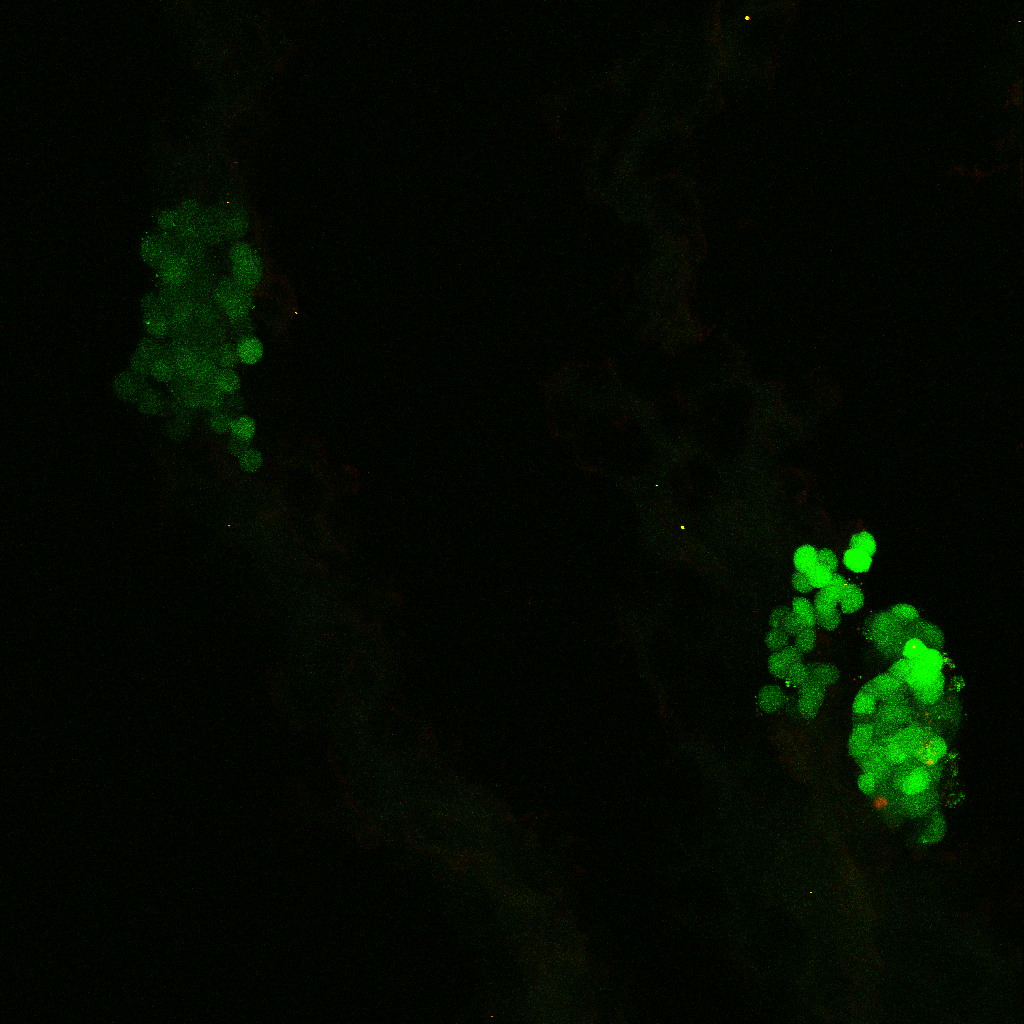

Supplement: S2 File — (ZIP) [file pone.0317350.s002.zip › S5_Image Folder/012022/012022_7 days_0.5-5mM RU-SPS_Disk 210707_20x_S1_Maximum intensity projection_b0v0t0z0c0-2x0-1024y0-1024.tif]

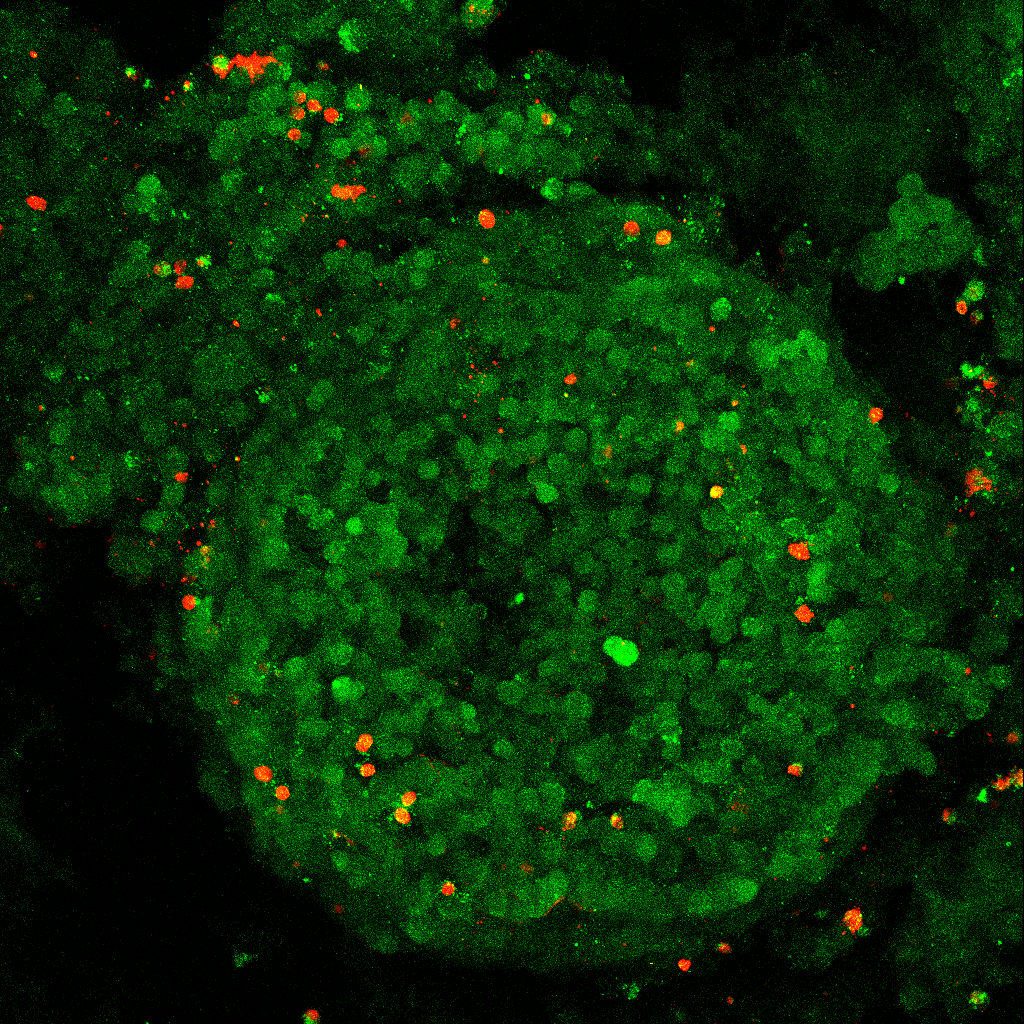

Supplement: S2 File — (ZIP) [file pone.0317350.s002.zip › S5_Image Folder/012022/012022_7 days_0.5-5mM RU-SPS_Disk 210707_20x_S2_Maximum intensity projection_b0v0t0z0c0-2x0-1024y0-1024.tif]

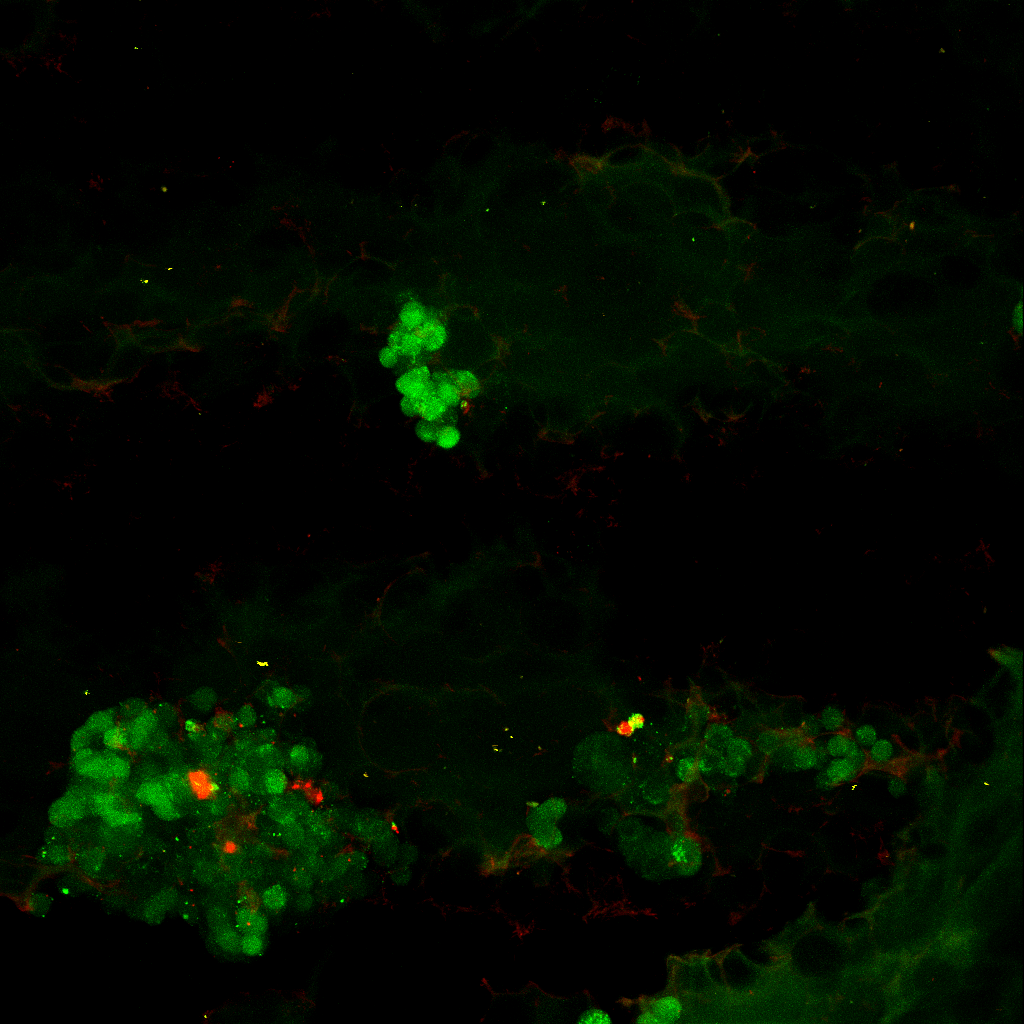

Supplement: S2 File — (ZIP) [file pone.0317350.s002.zip › S5_Image Folder/012022/012022_7 days_0.5-5mM RU-SPS_Disk 210707_20x_S3_Maximum intensity projection_b0v0t0z0c0-2x0-1024y0-1024.tif]

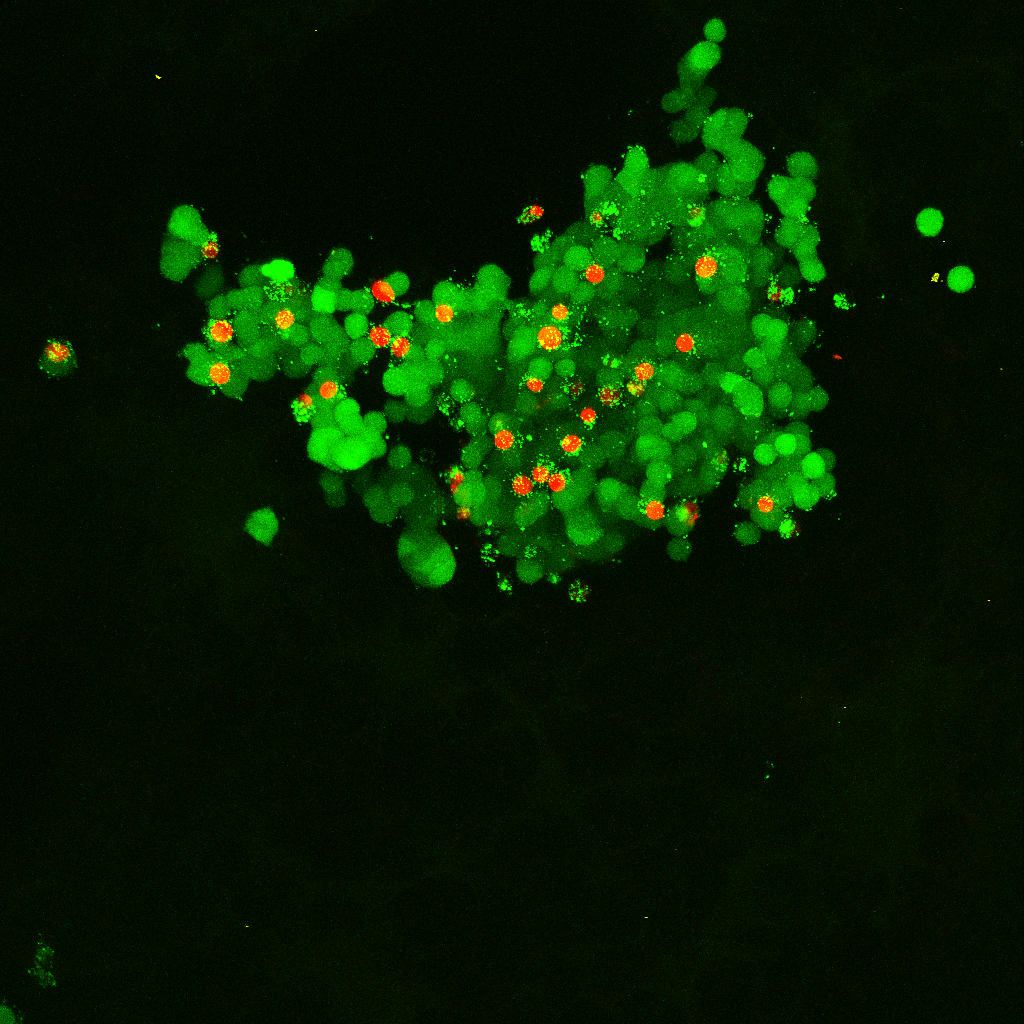

Supplement: S2 File — (ZIP) [file pone.0317350.s002.zip › S5_Image Folder/011922/011922_7 days_1.5-15mM RU-SPS_Disk 210707_20x_S4_Maximum intensity projection_b0v0t0z0c0-2x0-1024y0-1024.tif]

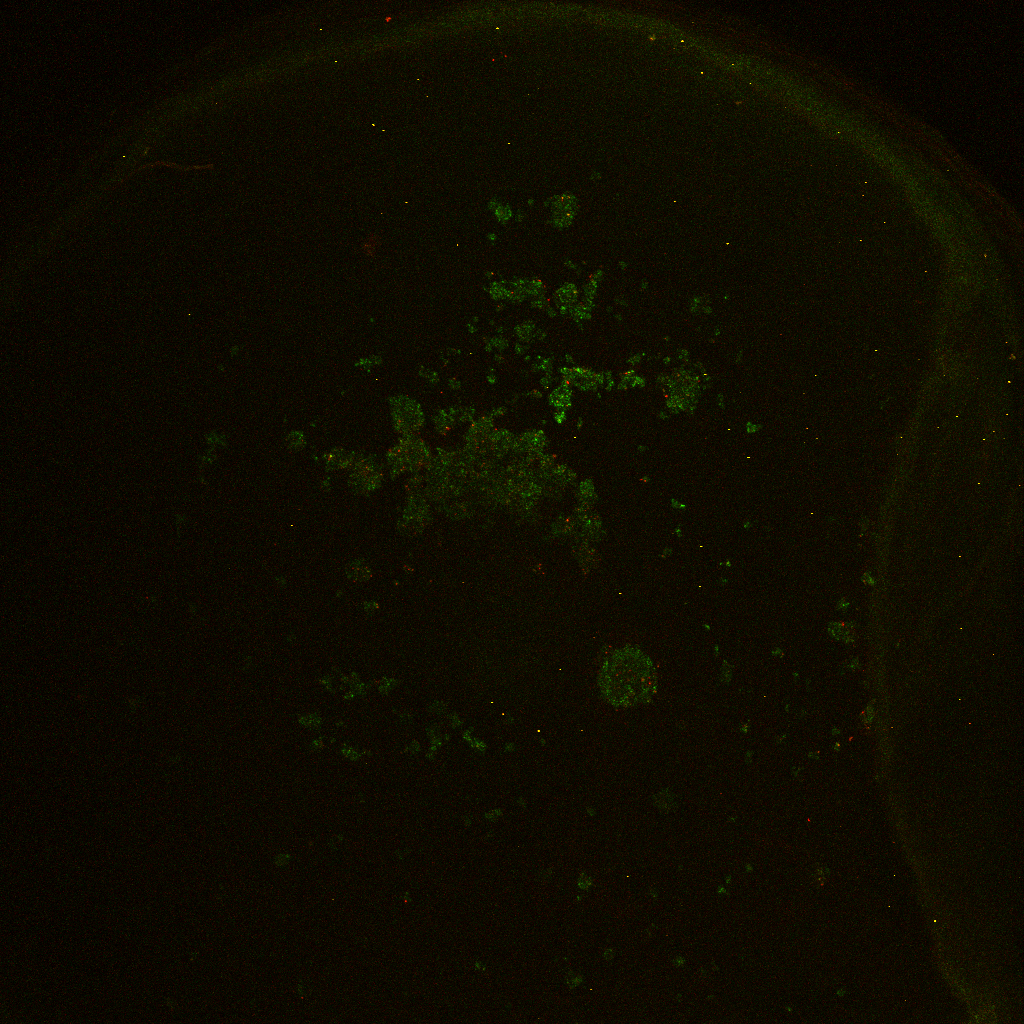

Supplement: S2 File — (ZIP) [file pone.0317350.s002.zip › S5_Image Folder/012122/012122_7 days_1.0-10mM RU-SPS_Disk 210707_2_5x_S3_Maximum intensity projection_b0v0t0z0c0-3x0-1024y0-1024.tif]

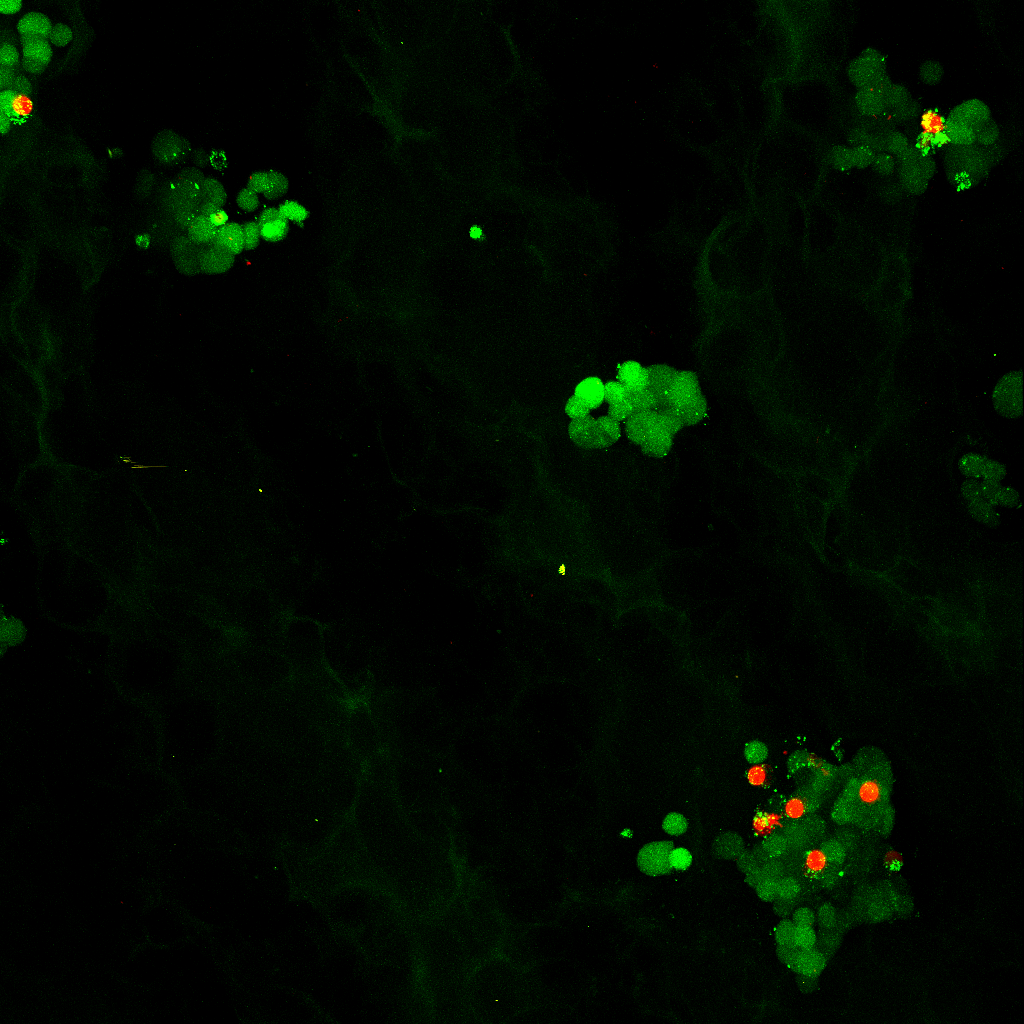

Supplement: S2 File — (ZIP) [file pone.0317350.s002.zip › S5_Image Folder/012122/012122_7 days_1.0-10mM RU-SPS_Disk 210707_20x_S2_Maximum intensity projection_b0v0t0z0c0-2x0-1024y0-1024.tif]

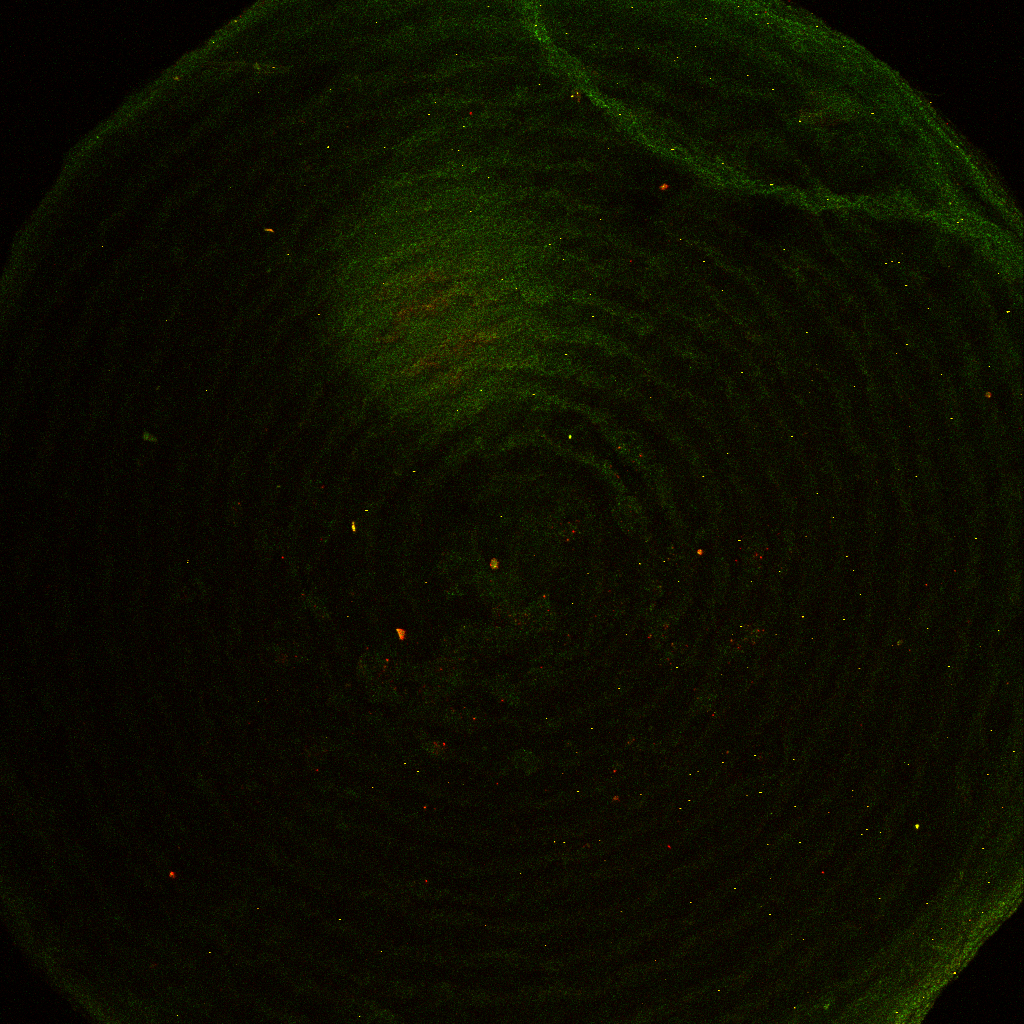

Supplement: S2 File — (ZIP) [file pone.0317350.s002.zip › S5_Image Folder/012022/012022_7 days_0.5-5mM RU-SPS_Disk 210707_2_5x_S4_Maximum intensity projection_b0v0t0z0c0-3x0-1024y0-1024.tif]

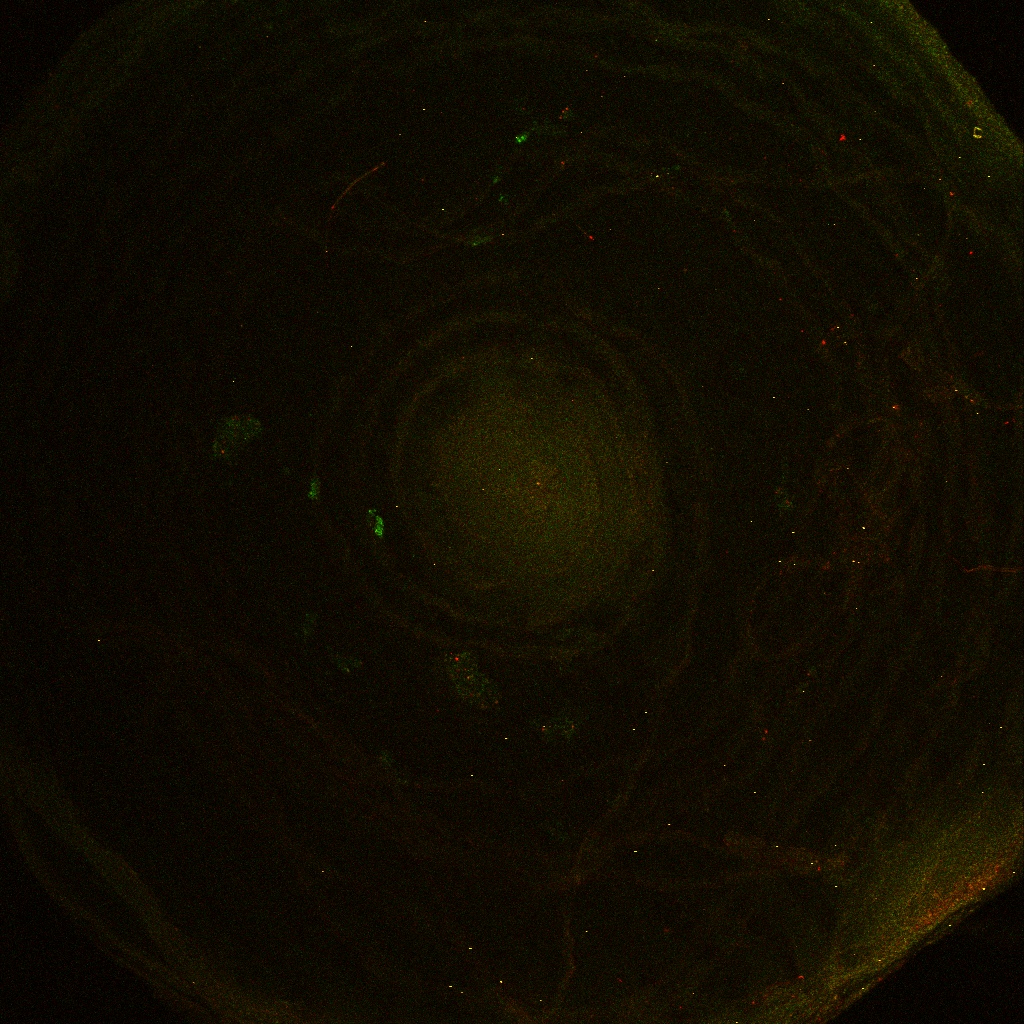

Supplement: S2 File — (ZIP) [file pone.0317350.s002.zip › S5_Image Folder/012022/012022_7 days_0.5-5mM RU-SPS_Disk 210707_2_5x_S1_Maximum intensity projection_b0v0t0z0c0-3x0-1024y0-1024.tif]

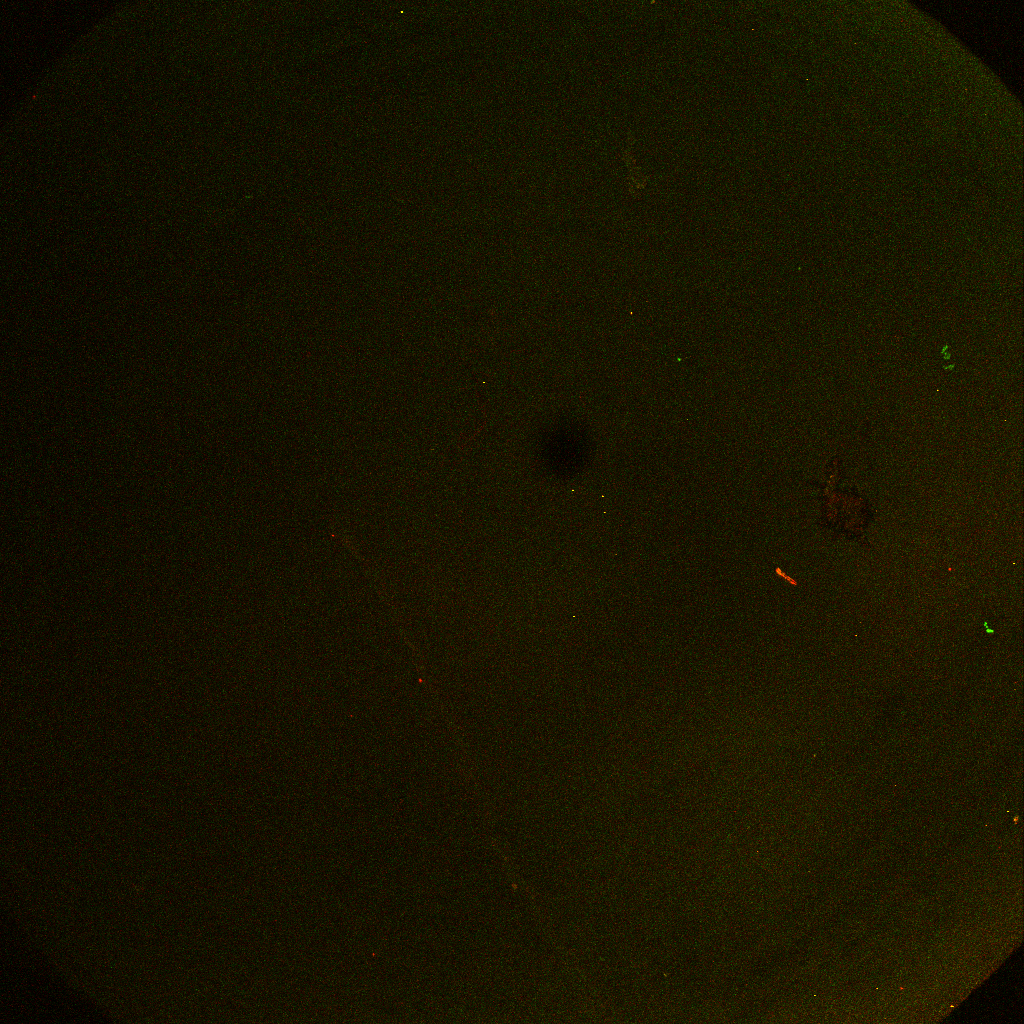

Supplement: S2 File — (ZIP) [file pone.0317350.s002.zip › S5_Image Folder/011922/011922_7 days_1.5-15mM RU-SPS_Disk 210707_2_5x_S1_Maximum intensity projection_b0v0t0z0c0-3x0-1024y0-1024.tif]

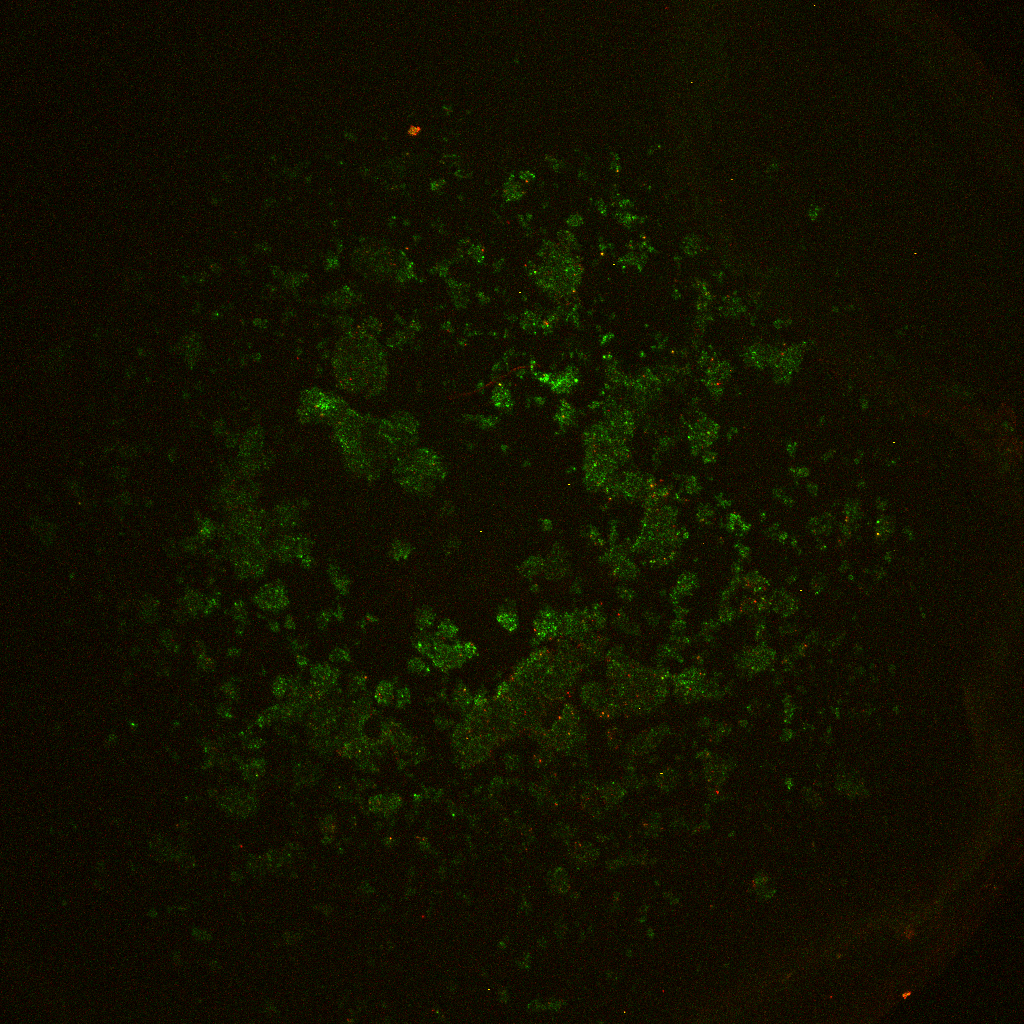

Supplement: S2 File — (ZIP) [file pone.0317350.s002.zip › S5_Image Folder/012122/012122_7 days_1.0-10mM RU-SPS_Disk 210707_2_5x_S1_Maximum intensity projection_b0v0t0z0c0-3x0-1024y0-1024.tif]

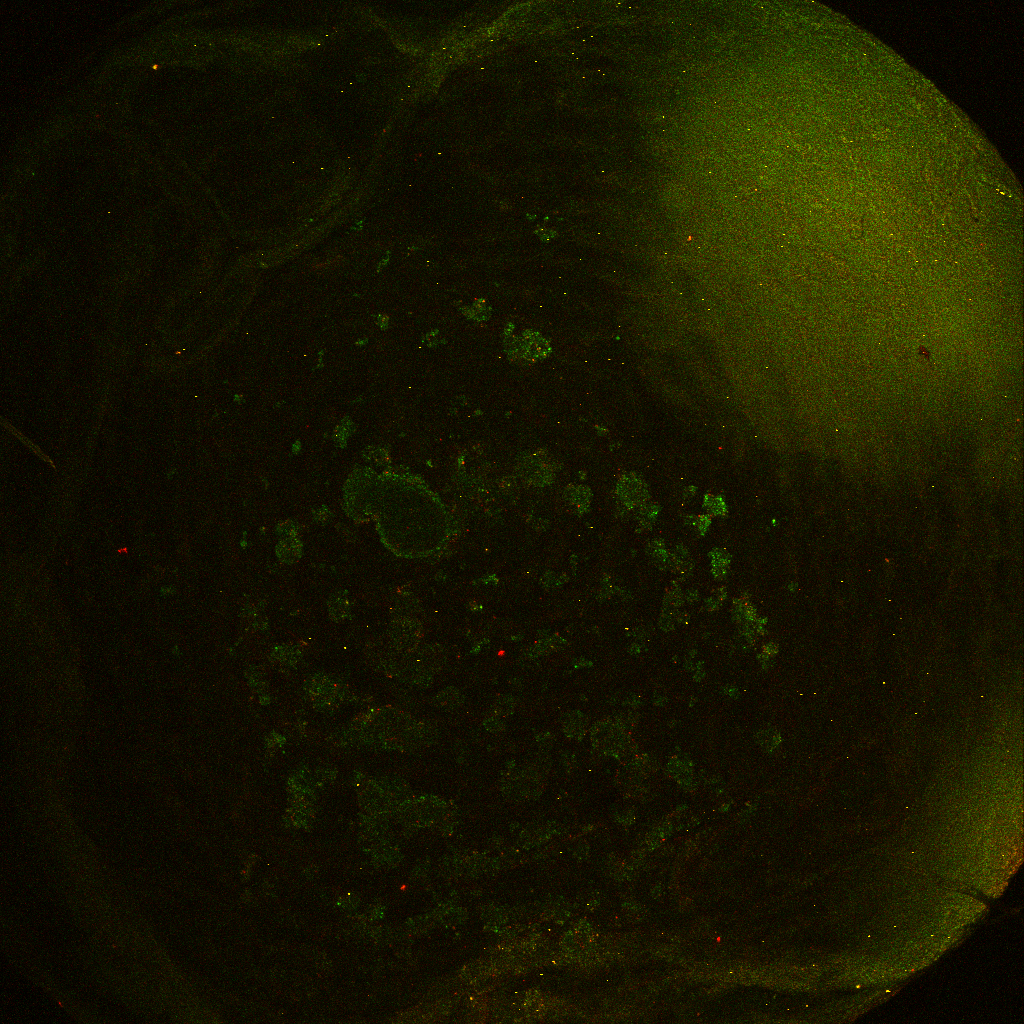

Supplement: S2 File — (ZIP) [file pone.0317350.s002.zip › S5_Image Folder/012022/012022_7 days_0.5-5mM RU-SPS_Disk 210707_2_5x_S2_Maximum intensity projection_b0v0t0z0c0-3x0-1024y0-1024.tif]

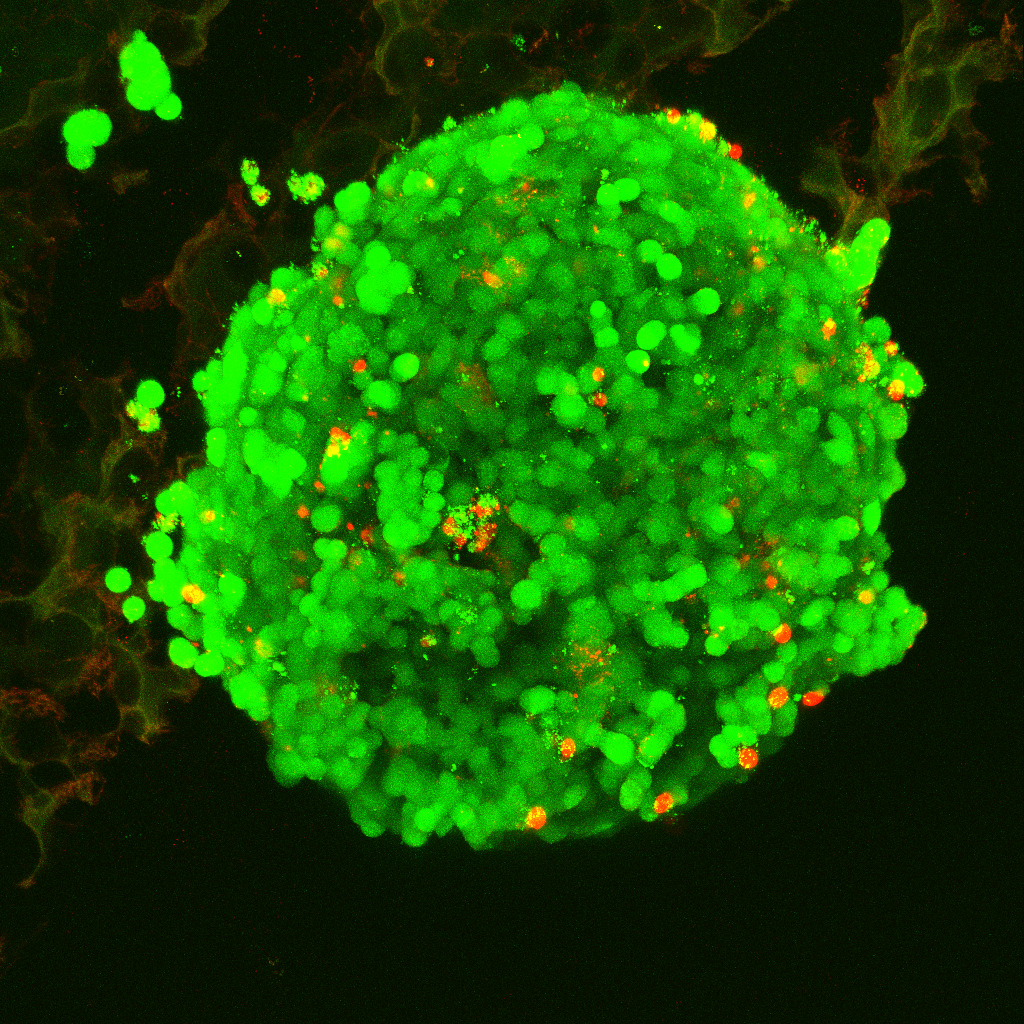

Supplement: S2 File — (ZIP) [file pone.0317350.s002.zip › S5_Image Folder/011922/011922_7 days_1.5-15mM RU-SPS_Disk 210707_20x_S2_org_Maximum intensity projection_b0v0t0z0c0-2x0-1024y0-1024.tif]

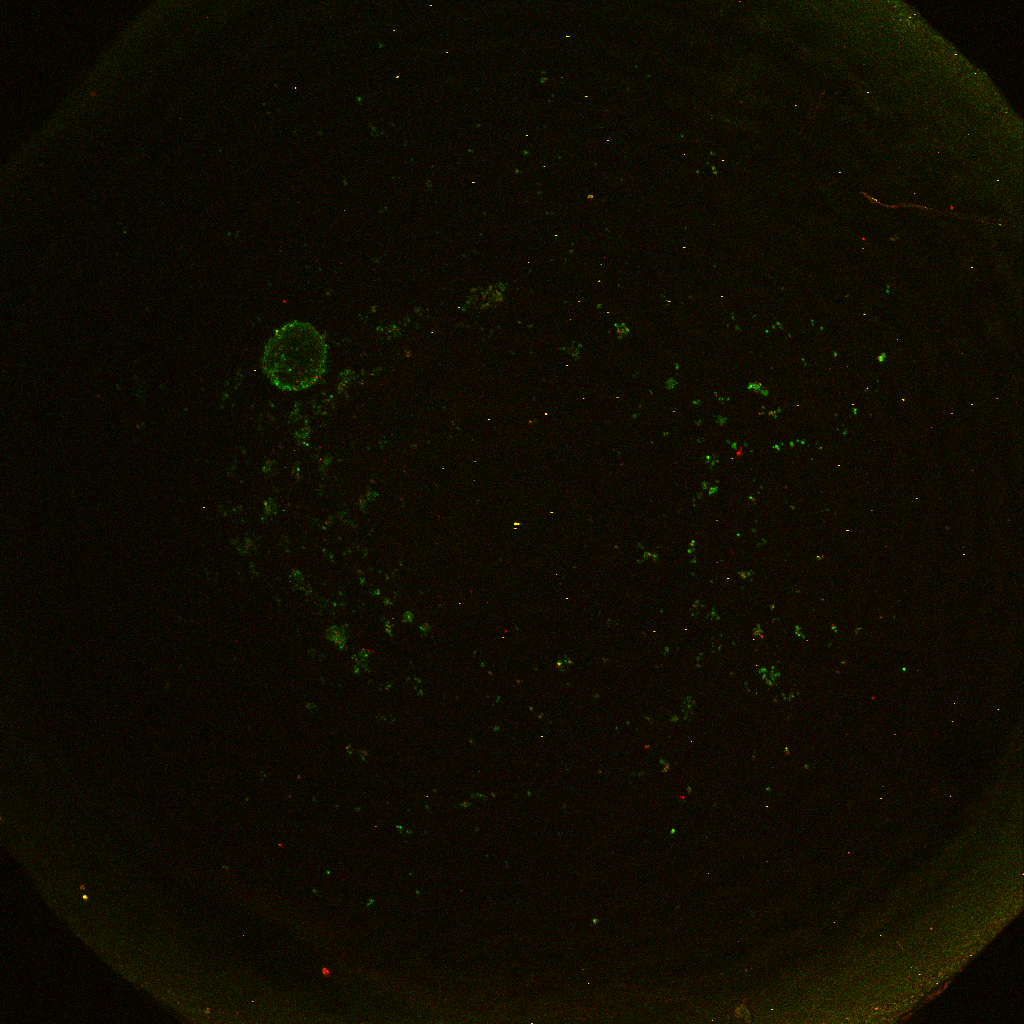

Supplement: S2 File — (ZIP) [file pone.0317350.s002.zip › S5_Image Folder/011922/011922_7 days_1.5-15mM RU-SPS_Disk 210707_2_5x_S2_Maximum intensity projection_b0v0t0z0c0-3x0-1024y0-1024.tif]

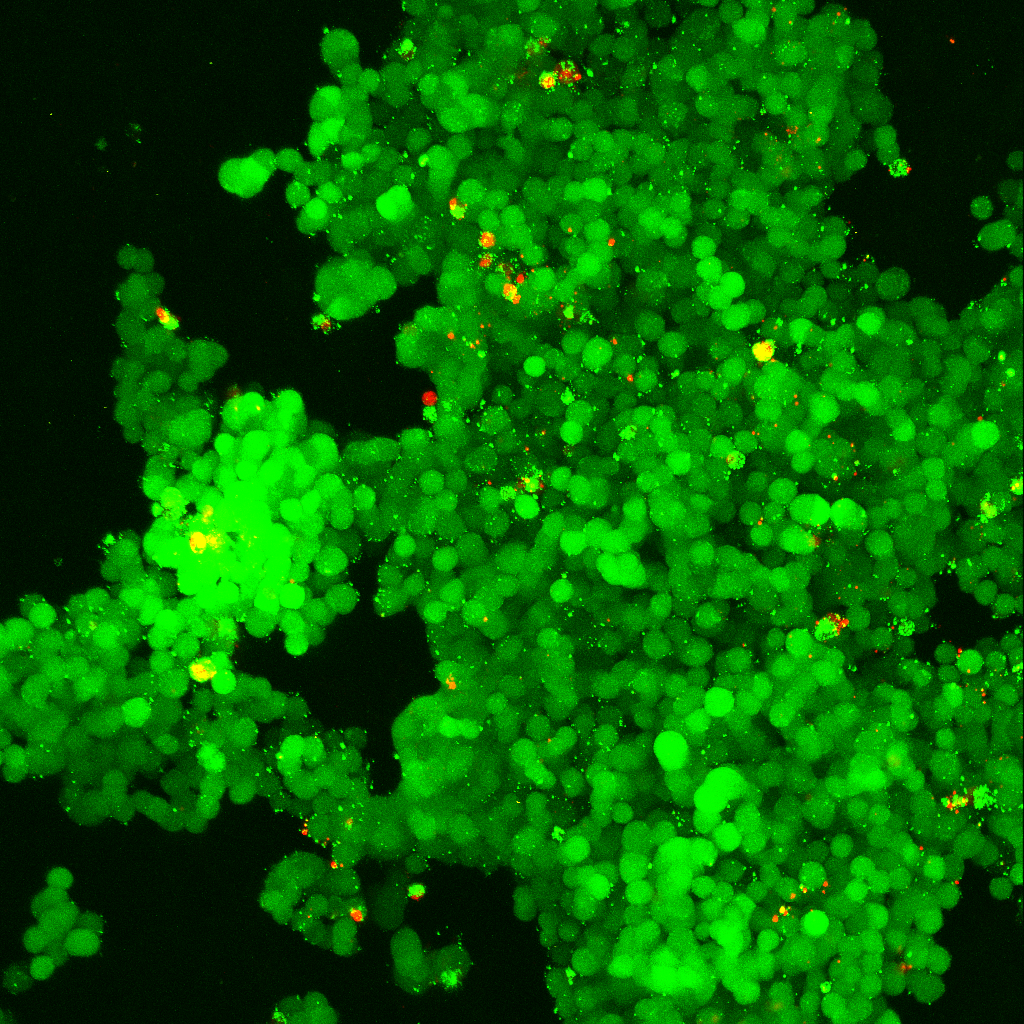

Supplement: S2 File — (ZIP) [file pone.0317350.s002.zip › S5_Image Folder/012122/012122_7 days_1.0-10mM RU-SPS_Disk 210707_20x_S1_Maximum intensity projection_b0v0t0z0c0-2x0-1024y0-1024.tif]

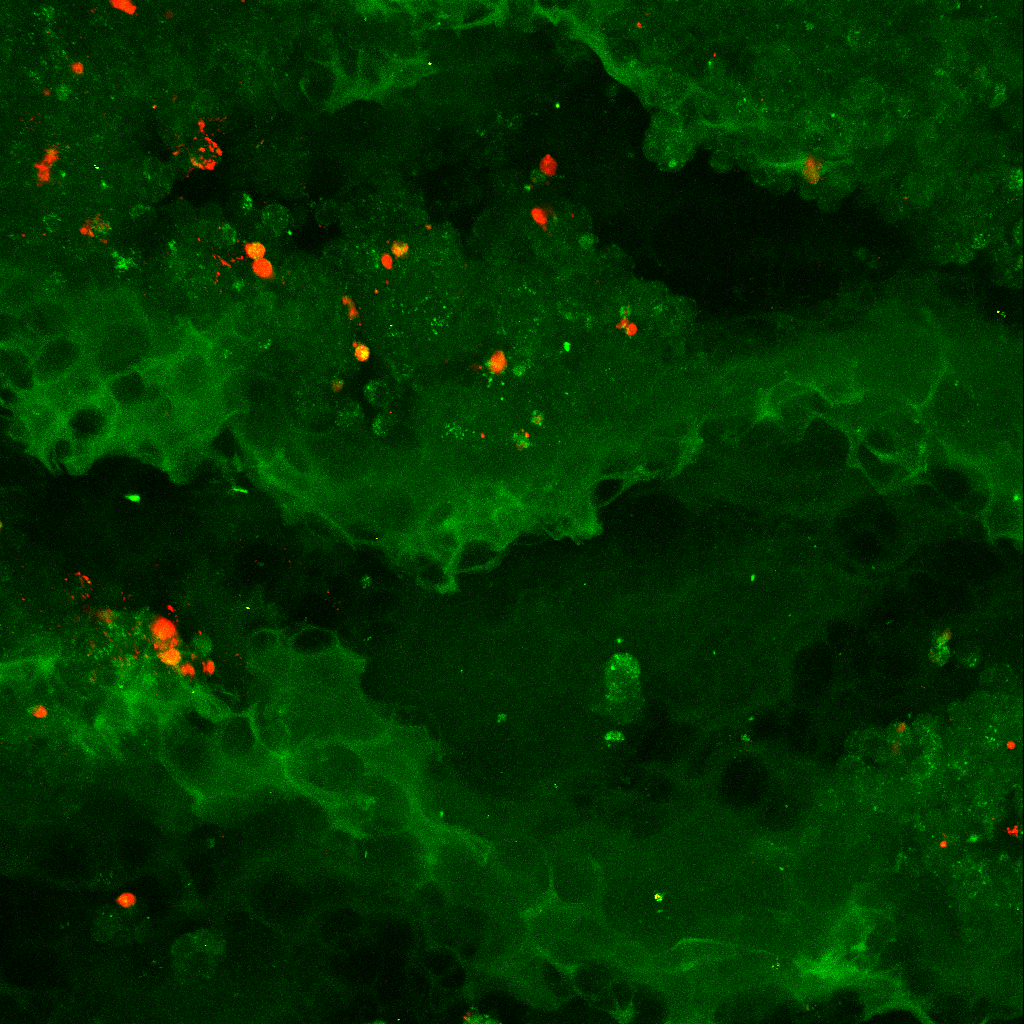

Supplement: S2 File — (ZIP) [file pone.0317350.s002.zip › S5_Image Folder/012022/012022_7 days_0.5-5mM RU-SPS_Disk 210707_20x_S4_Maximum intensity projection_b0v0t0z0c0-2x0-1024y0-1024.tif]

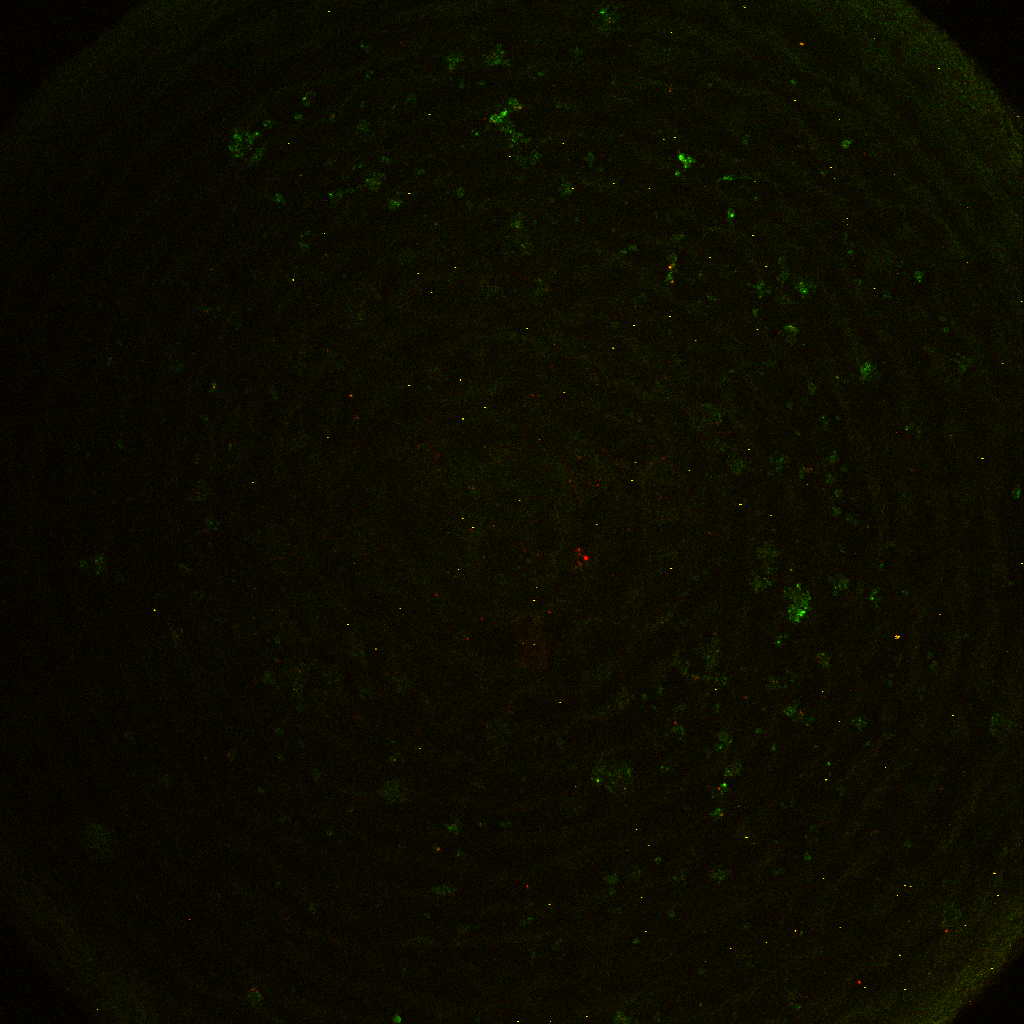

Supplement: S2 File — (ZIP) [file pone.0317350.s002.zip › S5_Image Folder/012122/012122_7 days_1.0-10mM RU-SPS_Disk 210707_2_5x_S2_Maximum intensity projection_b0v0t0z0c0-3x0-1024y0-1024.tif]

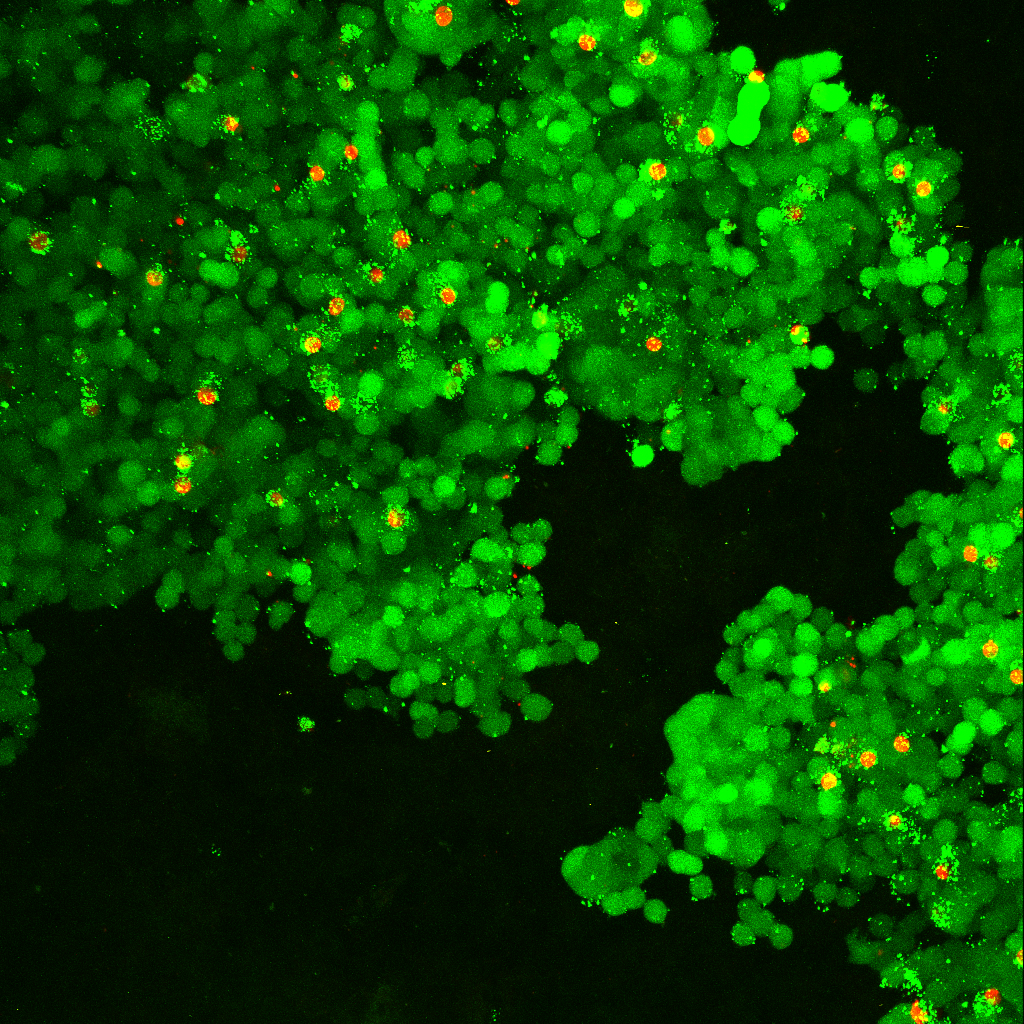

Supplement: S2 File — (ZIP) [file pone.0317350.s002.zip › S5_Image Folder/012122/012122_7 days_1.0-10mM RU-SPS_Disk 210707_20x_S3_Maximum intensity projection_b0v0t0z0c0-2x0-1024y0-1024.tif]

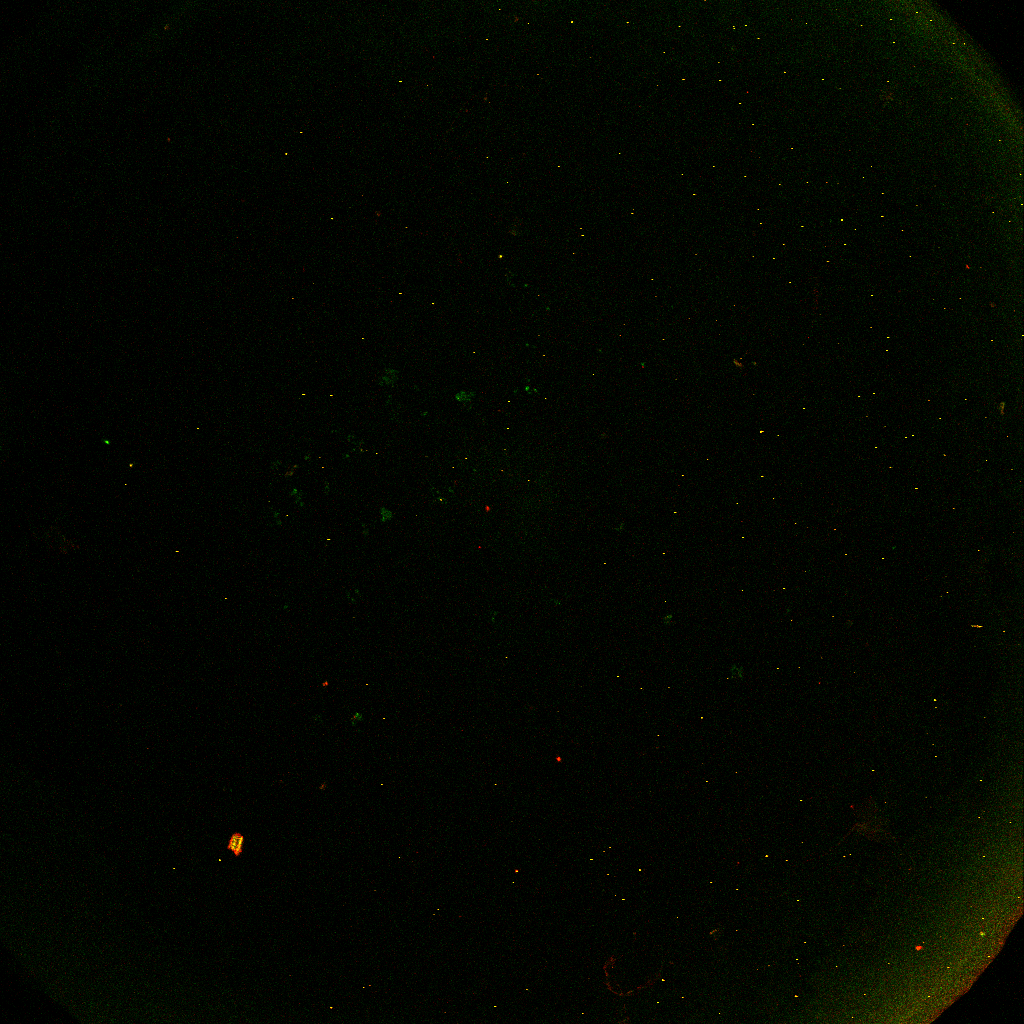

Supplement: S2 File — (ZIP) [file pone.0317350.s002.zip › S5_Image Folder/011922/011922_7 days_1.5-15mM RU-SPS_Disk 210707_2_5x_S3_Maximum intensity projection_b0v0t0z0c0-3x0-1024y0-1024.tif]

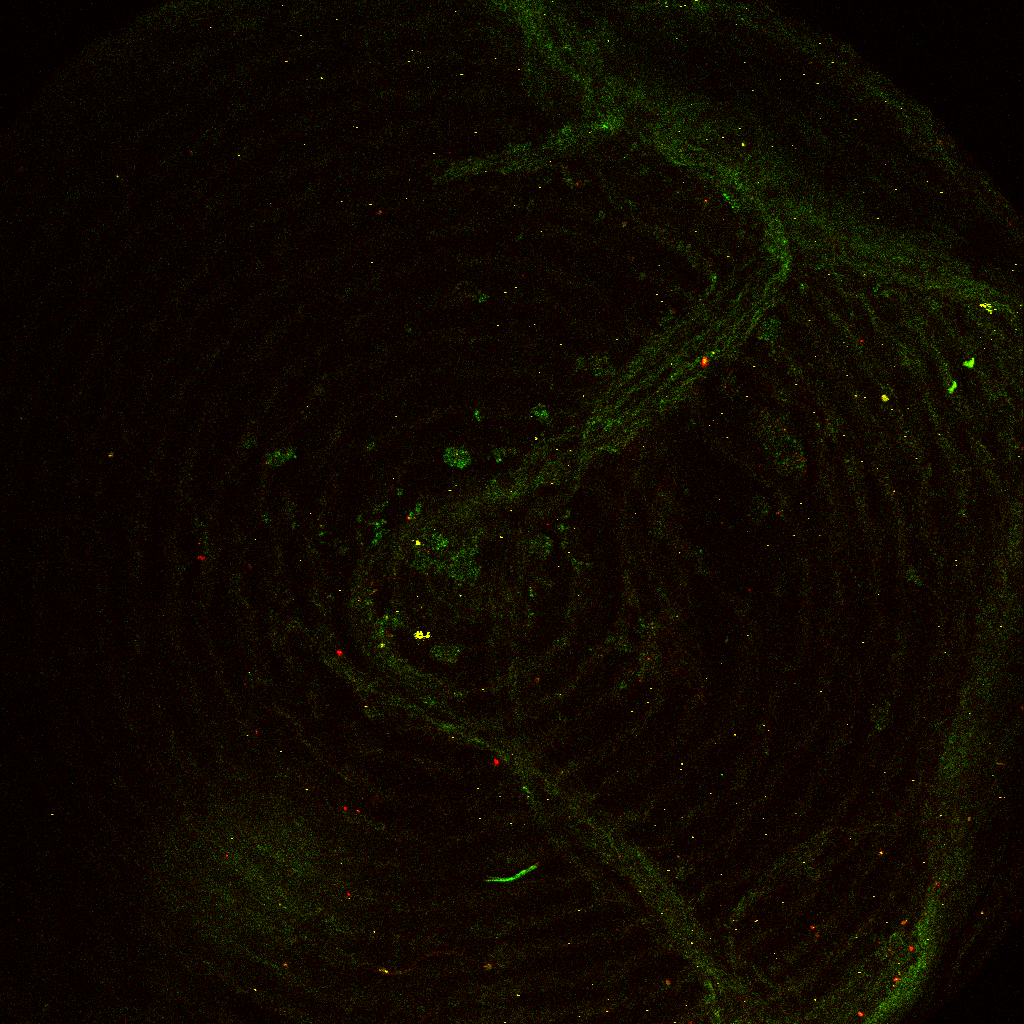

Supplement: S2 File — (ZIP) [file pone.0317350.s002.zip › S5_Image Folder/012022/012022_7 days_0.5-5mM RU-SPS_Disk 210707_2_5x_S3_Maximum intensity projection_b0v0t0z0c0-3x0-1024y0-1024.tif]

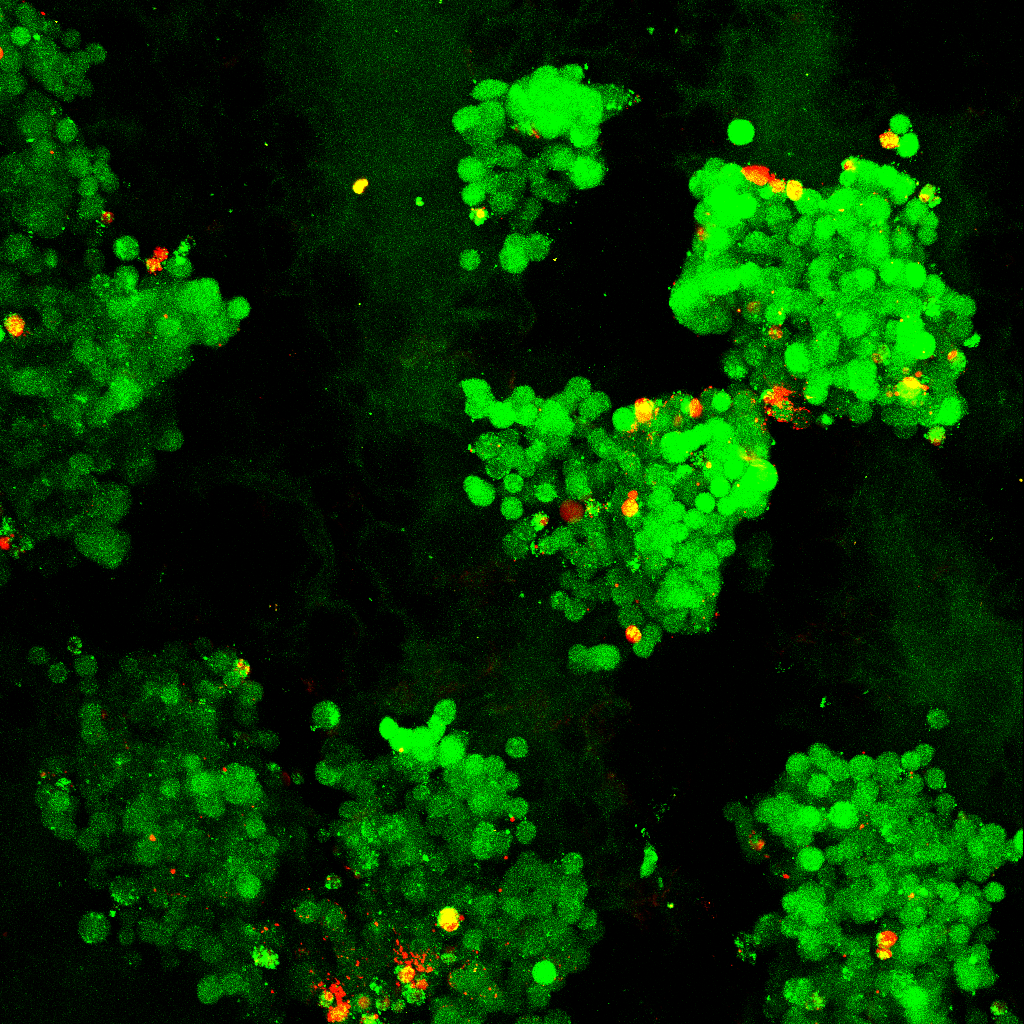

Supplement: S2 File — (ZIP) [file pone.0317350.s002.zip › S5_Image Folder/012022/012022_7 days_0.5-5mM RU-SPS_Disk 210707_20x_S2_B_Maximum intensity projection_b0v0t0z0c0-2x0-1024y0-1024.tif]

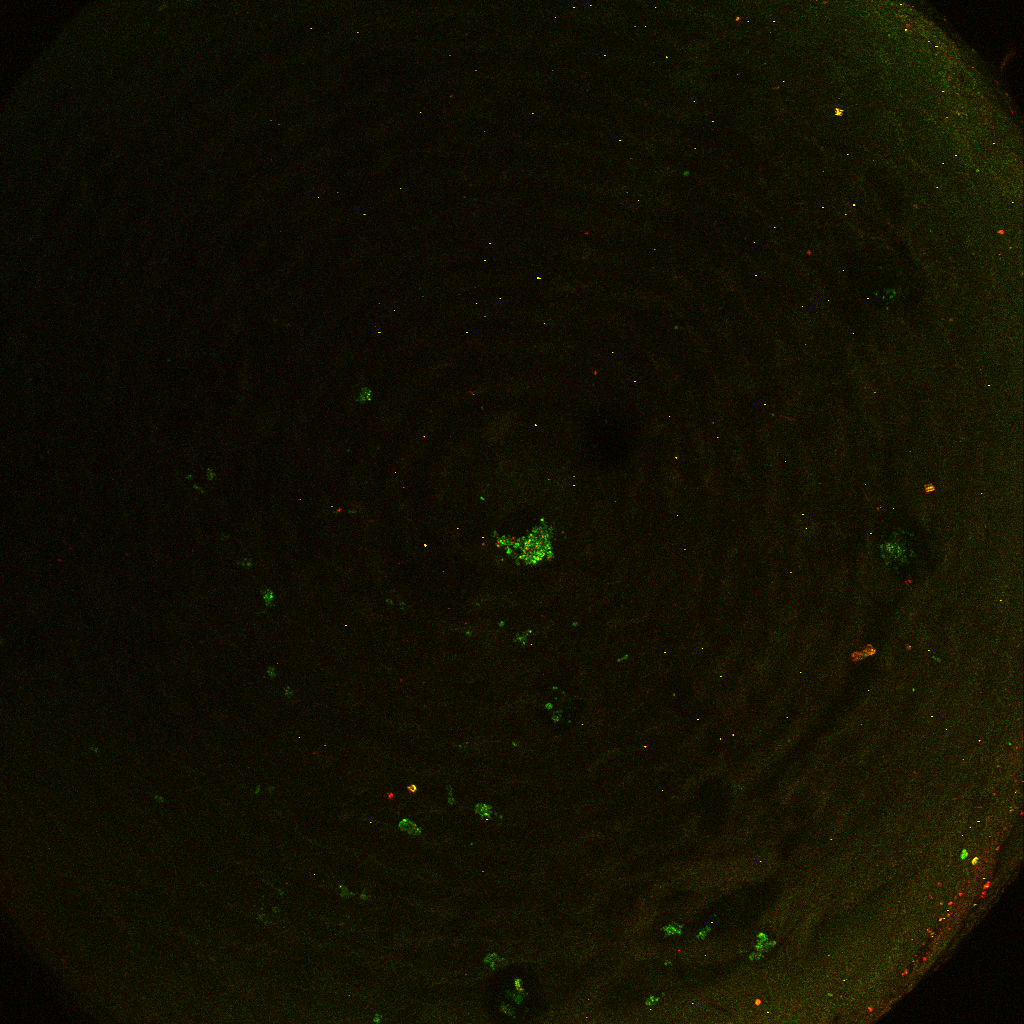

Supplement: S2 File — (ZIP) [file pone.0317350.s002.zip › S5_Image Folder/011922/011922_7 days_1.5-15mM RU-SPS_Disk 210707_2_5x_S4_Maximum intensity projection_b0v0t0z0c0-3x0-1024y0-1024.tif]

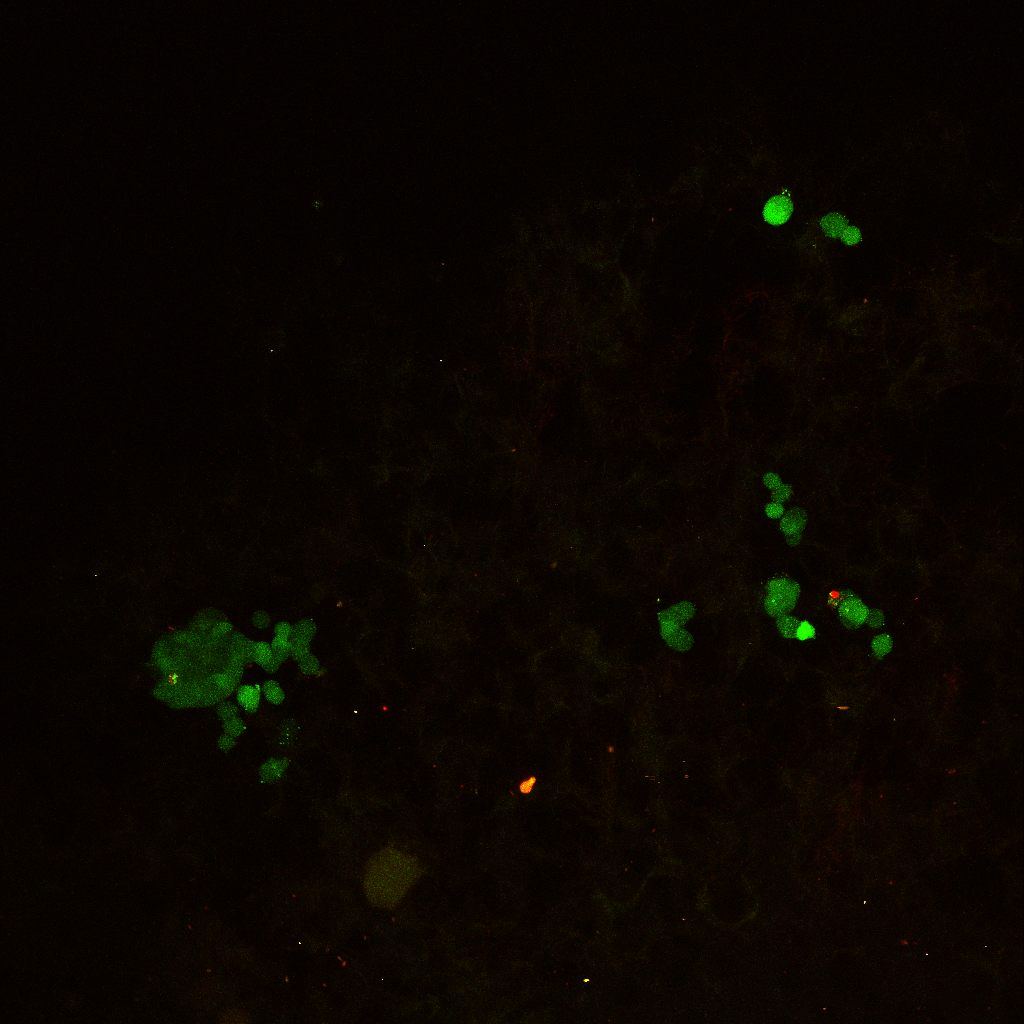

Supplement: S2 File — (ZIP) [file pone.0317350.s002.zip › S5_Image Folder/011922/011922_7 days_1.5-15mM RU-SPS_Disk 210707_20x_S3_Maximum intensity projection_b0v0t0z0c0-2x0-1024y0-1024.tif]
