# Supplementary material for: FRESH extrusion 3D printing of type-1 collagen hydrogels photocrosslinked using ruthenium
Source: PLoS One. 2025 Jan 10;20(1):e0317350. doi: 10.1371/journal.pone.0317350 (PMC11723599; doi:10.1371/journal.pone.0317350)
Supplement: S1 Protocol — (DOCX) [file pone.0317350.s005.docx]

# **Protocol for FRESH extrusion 3D printing of Type-1 collagen hydrogels photocrosslinked using Ruthenium**

# Richard C. Steiner, PhD ^1,2,†^, Jack T. Buchen, ^1,2,†^, Evan R. Phillips, PhD^1, 3^, Christopher R. Fellin, PhD ^1,2^, Xiaoning Yuan MD, PhD ^2^, Shailly H. Jariwala, PhD ^1,2,*^

# ^1^ The Henry M. Jackson Foundation for the Advancement of Military Medicine, Inc., 6720A Rockledge Drive, Suite 100, Bethesda, MD 20817, USA

# ^2^ The Center for Rehabilitation Sciences Research, Department of Physical Medicine and Rehabilitation, Uniformed Services University of Health Sciences, 4301 Jones Bridge Rd, Bethesda, MD 20814, USA

# ^3^ CytoSorbents Medical Inc., 305 College Road East, Princeton, New Jersey, 08540, USA

# ^†^ These authors contributed equally to this work

# ^*^ Corresponding author: shailly.jariwala.ctr@usuhs.edu

# **Funding:** This study was funded by the Center for Rehabilitation Sciences Research (CRSR) In-House Laboratory Independent Research (ILIR), Department of Physical Medicine and Rehabilitation, Uniformed Services University, Bethesda, MD, USA (award # HU00012320007).

# **Competing interests:** The authors confirm that there are no known conflicts of interest associated with this publication.

**Data availability:** Data can be made available upon request.

**Associated content:** DOI for accompanying article in Plos ONE to be included once published

## Preparing The LifeSupport:

**Materials:**

- Advanced Biomatrix LifeSupport lyophilized gelatin microspheres (2 gram); Catalog No. 5244-8GM
- Fisher Scientific Gibco™ 1X PBS, pH 7.4; Catalog No 10-010-049

# Corning® 50 mL centrifuge tubes

# Kimwipes® disposable wipers

**Equipment:**

- Eppendorf Centrifuge 5810 R 15amp
- Heidolph Instruments Vortex D-91126 Schwabach REAX top 541-10000-01-1
- 40mm x 25mm glass dish
- Fisherbrand™ Semimicro Spatula with One Tapered End, One Rounded End 14-374

1. Prepare the centrifuge by cooling the temperature to 4℃.
2. Prepare 35ml of 1X Phosphate Buffered saline (PBS), and refrigerate until temperature reaches 4℃.
3. Retrieve the Advance Biomatrix LifeSupport, which is stored at room temperature.
4. Prepare an empty 50ml conical tube for mixing LifeSupport. Separate the LifeSupport powder into two 1g aliquots, each in a separate 50ml conical tube.
5. To each of the 1g aliquots of LifeSupport, add 17.5ml of 4℃ PBS.
6. For each conical tube, shake vigorously for 1 minute by A) shaking horizontally along the length of the tube, and B) tapping the end of the tube against the edge of the table. Continue until the gelatin has been uniformly mixed, and there’re no longer large clumps clinging to the walls of the tube.
7. Place the conical tubes containing LifeSupport into a 4℃ refrigerator for 45 minutes. This step allows the LifeSupport to fully rehydrate, and ensures the mixture is at an adequate temperature.
8. Vortex each conical tube for 45 seconds.
9. Centrifuge the conical tubes at 2500g for 5 minutes.
10. Remove conical tubes from the centrifuge, open the caps, and pour off the supernatant. Absorb the remaining supernatant with Kimwipes (low-linting) to remove as much liquid as possible.
11. Recap and shake the conical tubes again. For each conical tube, shake vigorously for 1 minute by A) shaking horizontally along the length of the tube, and B) tapping the end of the tube against the edge of the table. Continue until the gelatin has been uniformly mixed, and there are no longer large clumps clinging to the walls of the tube.
12. Vortex each conical tube for 45 seconds.
13. Centrifuge the conical tubes again at 2500g for 5 minutes.
14. Pour off or absorb any remaining supernatant with a Kim wipe.
15. Prepare a 40 x 25mm glass dish.
16. Uncap the first conical tube, and invert the tube into the glass dish so that it’s upside down with the open end inside the glass dish. Tap the tube up and down vertically until the gelatin LifeSupport dislodges and fills the glass dish.
17. Remove the conical tube from the dish. Using a small spatula, scoop the remaining LifeSupport from the walls of the conical tube, being careful not to introduce new air bubbles. Any LifeSupport that is scooped out of the conical tube should be placed on top of and in the center of the LifeSupport that is sitting in the glass dish.
18. Carefully but forcefully tap the glass dish flat against a table to get the LifeSupport to spread out and fill the base of the dish. If small air bubbles form during this process, use the spatula to carefully scoop them out, and then tap the LifeSupport against the table again to fill the void.
19. Uncap the second conical tube, and carefully scoop the LifeSupport out, careful not to create air bubbles. Place the LifeSupport from the second tube on top of the LifeSupport from the first tube. Again, tap against the table to disperse the LifeSupport evenly. Use the spatula to remove any large air bubbles that form.
20. LifeSupport should achieve a vaseline-like consistency, with no flow of material from side-to-side when tilted.
21. The LifeSupport can be stored at 4ºC for up to 60 minutes but should be used as quickly as possible to obtain the best results.

# Printing:

**Ink preparation**

Materials:

- Advanced Biomatrix Lifeink 240 Acidified Collagen Bioink 35mg/ml (5 ml); Catalog No. 5267

Equipment:

- Allevi Plastic Syringes (5cc); Catalog No. SKU-PSYR5
- Eppendorf Centrifuge 5810 R 15amp
- 40 x 25mm glass dish
- 30 gage, 1-inch blunt end, lavender, FisnarⓇ; Catalog No. 8001104
- Allevi 3 systems, SN. 7c1057c
- Sundee glue tape

Software:

- Slic3r
- Repitier
- Autodesk Fusion, Shapr3D, or TinkerCAD

1. Commercially available 5cc syringes designed for Allevi 3 systems 3D printers were used to hold and disperse LifeInk 240 during 3D printing. 5cc syringes were initially prepared by removing the plunger cap from the plunger and placing it at the bottom of the syringe till it is in contact with the syringe neck.
2. Using a syringe female-female luer lock adapter, we transfer 4 ml of lifeink 240 ink from our stock 5cc syringe to the 5cc syringes.
3. Elimination of air bubbles and gaps in the ink was accomplished by capping the neck of 5cc syringes using lure locks and placing the neck of the syringe down in the centrifuge.
4. Centrifuge parameters were set to 4℃ and 2000G force for 5 min spin.
5. To remove any final air bubbles, we would insert another syringe with a 30 gage needle to suck out any air pockets and move the plunger cap down into the empty space carefully without ejecting ink out of the syringe.
6. The prepared syringe was stored in a 4℃ fridge until printing.

**3D print file**

1. 3D files can be designed using standard computer-aided-design (CAD) software. Popular options include AutoCAD, Solidworks, Autodesk Fusion, Shapr3D, etc. Any CAD software that can adequately meet your design needs and can export files as .stl or .obj is acceptable.
   1. 3D files were designed in Autodesk Fusion, Shapr3D, or TinkerCAD. Files were exported in .stl file format.
2. After 3D files have been created, they need to be “sliced.” Slicing is the process by which the 3D file is translated into a G-code file. G-code is a software programming language used to control 3D machines, such as CNC machines and 3D printers. The Allevi 3 3D Bioprinter used in this process accepts G-code files. Popular slicing software includes UltiMaker Cura, PrusaSlicer, Slic3r, Repetier, etc.

Files were sliced using Slic3r and Repetier.

1. To slice a 3D CAD file, open Repetier.
2. Select “Config”, and select the standard printer configuration.
3. Click “Load,” then upload your .stl 3D file.
4. Under “Object Placement,” assign extruder 0 to the “Object Group 1.”
5. Click “Slicer.” From the dropdown menu titled “Slicer:” select “Slic3r.”
6. If desired, select “Configuration” to choose your specific slicing parameters (see below), then save and return to the Repetier window.
7. Ensure that “Extruder 1:” selection lists your extruder that the bioink will be extruded from.
8. Click “Slice with Slic3r.” Wait for the Slic3r window to pop up.
9. Click “Export G-code…” Save the Gcode. You will upload it to the Allevi 3 Bioprinter in the next section.

**Slicing Parameters:**

For our purposes in designing a nerve guide conduit, we used the following parameters:

**50% Infill** (This prevents the object from being too dense and overfilled)

**Rectilinear Infill** (This is the pattern of infill inside your print)

**150% Overlap** (This ensures that each individual layer and line is properly connected to the last, allowing for cohesion of layers during crosslinking)

**No Perimeter** (Typically slicing software will allow you to print additional perimeters around your design with a different set of parameters than the rest of the design. We avoided this for two reasons: 1) Additional perimeters were not factored into our design specifications, thus adding perimeters after the fact would increase the size and deform the shape. 2) In our extensive testing, we found that perimeters, with our particular collagen bioink, tended to shear and separate from the rest of the print)

**0.10mm Layer Height** (Setting the layer height shorter than the inner diameter of the needle allowed us to force additional vertical overlap, thus creating a well-bonded and cohesive object)

**0.17mm Extruder Width** (The extruder width is traditionally set at or slightly larger than the inner diameter of the extruder nozzle. In this situation, the inner diameter of our needle was 0.15mm, but through experimentation and testing, we identified 0.17mm to be the best extruder width)

**6 mm/s print speed** (This is the tool speed at which the extruder moves along it’s path. For our purposes, we identified 6mm/s to be the best print speed, both for print quality and to prevent excessive drag interference.)

**Allevi 3 systems prep**

1. Allevi 3 systems should be turned on and connected to a WiFi server without interruption during the printing process.
2. Make sure working room environmental conditions read to between 17-22℃ with minimal surface vibrations for optimal printing.
3. Using an empty 40 x 25mm glass dish, center the dish on the print stage then mark the edges of the dish onto the print stage with a marker. Using an empty 5cc syringe with a 1 inch, 30 gauge needle attached, position the needle into the printer head and lock in place with the air compression hose.
4. On the Allevi interface, we use the directional controls (x,y, and z-axis) to position the tip of the needle to the bottom center of the 40 x 25mm glass dish. We position the tip of the needle so that there is only 0.1mm z-spacing between the needle tip and the glass dish surface. If the position of the needle tip relative to the glass dish is correct and acceptable to the print dimensions, then using the Allevi interface move the print stage on the z-axis down from the needle tip by 25 mm. Remove the empty syringe from the print head and the glass dish from the print stage.
5. Set the printer head parameters on the interface screen to the following: Ink temp 4℃, air pressure of 50psi.
6. Collect the prepared 5cc of LifeInk from the 4℃ fridge. Place into the syringe slot on the printer head and securely attach and lock the air compression hose to the top of the syringe.
7. Calibrate the extrusion properties of the ink by manually extruding the ink through the length of the needle and observing the rate of droplet formation at the needle tip. Manually extrude several times to clear the line of air bubbles. Make sure to wipe the needle tip with Kim wipe after testing to ensure no residual dried ink will interfere with droplet formation and ink flow.
8. Upload the Gcode file for the desired print to the Allevi server using the Allevi interface. Ensure the Gcode is selected in the interface screen before printing.
9. Collect the prepared lifesupport dish from the 4℃ fridge. Add double-sided Sundee glue tape to the bottom of the dish, then place the dish on the printer stage so that the edges of the dish align with the marks on the stage. Ensure the dish is adhered tightly to the stage.
10. Press the print button on the interface to begin printing.

# Crosslinking:

Materials:

# Millipore Sigma Tris(2,2′-bipyridyl)dichlororuthenium(II) hexahydrate; Catalog No 224758

# Millipore Sigma Sodium persulfate; Catalog NoS6172

- Fisher Scientific Gibco™ 1X PBS, pH 7.4; Catalog No 10-010-049

# Fisherbrand™ Premium Microcentrifuge Tubes: 2.0mL; Catalog No 05-408-138

- Aluminum foil
- Fisherbrand 10ml serological pipettes (2X)

Equipment:

- Heidolph Instruments Vortex D-91126 Schwabach REAX top 541-10000-01-1
- AmScope 3WX2 LED-6WD LED Spot Light with Two 6500K LED Attachments; Catalog Number YAG20201215
- 100-1000 µl pipette, Fisherbrand Elite NU09348 and pipette tips
- Drummond Scientific Co. Pipetaid XP
- Thermoscientific HERRAcell i150 Incubator

### Preparing the solutions:

**For 25/250 mM Ru/SPS Concentration:**

1. Prepare two 2.0ml microcentrifuge tubes by wrapping them in aluminum foil to prevent light leakage.
2. Label one tube Ru and the other SPS.
3. To each, add 1 ml of 1X PBS.
4. To the tube labeled Ru, add 0.0187g of Tris(2,2′-bipyridyl)dichlororuthenium(II) hexahydrate. Close the tube and ensure it is adequately wrapped in aluminum foil.
5. To the tube labeled SPS, add 0.0595g of Sodium persulfate. Close the tube and ensure it is adequately wrapped in aluminum foil.
6. For each tube, vortex for one minute to ensure that the mixture is uniformly distributed.

**For 50/500 mM Ru/SPS Concentration:**

1. Prepare two 2.0ml microcentrifuge tubes by wrapping them in aluminum foil to prevent light leakage.
2. Label one tube Ru and the other SPS.
3. To each, add 1 ml of 1X PBS.
4. To the tube labeled Ru, add 0.0374g of Tris(2,2′-bipyridyl)dichlororuthenium(II) hexahydrate. Close the tube and ensure it is adequately wrapped in aluminum foil.
5. To the tube labeled SPS, add 0.1191g of Sodium persulfate. Close the tube and ensure it is adequately wrapped in aluminum foil.
6. For each tube, vortex for one minute to ensure that the mixture is uniformly distributed.

**For 75/750 mM Ru/SPS Concentration:**

1. Prepare two 2.0ml microcentrifuge tubes by wrapping them in aluminum foil to prevent light leakage.
2. Label one tube Ru and the other SPS.
3. To each, add 1 ml of 1X PBS.
4. To the tube labeled Ru, add 0.0561g of Tris(2,2′-bipyridyl)dichlororuthenium(II) hexahydrate. Close the tube and ensure it is adequately wrapped in aluminum foil.
5. To the tube labeled SPS, add 0.1786g of Sodium persulfate. Close the tube and ensure it is adequately wrapped in aluminum foil.
6. For each tube, vortex for one minute to ensure that the mixture is uniformly distributed.

### Determining the Volume to add to different dish sizes:

Calculate the volume of the dish you used for printing, and multiply the volume of the dish by 2%. This will give you the volume of each solution you need to pipette into the dish for crosslinking.

*For Example: 40 x 25mm glass dish*

$$V=h\pi r^{2}$$

$$V=(25mm)\pi{(20mm)}^{2}$$

$$V=31.42ml$$

$$(31.42ml)(0.02)=0.6284ml$$

### Visible Light Crosslinking- Wavelength and Power Density:

We used an AmScope 3WX2 LED-6WD LED Spot Light with Two 6500K LED Attachments that was positioned approximately 75mm away from the sample.

The light produced by this system was 425nm, with a power density of 53.3$mW/cm^{2}$.

### Crosslinking the collagen Print:

1. Place a container of 500ml of 1X PBS in an incubator or water bath at 37ºC until it comes to temperature.
2. Place the 40 x 25mm glass dish containing the LifeSupport and completed Lifeink 240 collagen print in an incubator at 37ºC for 45 minutes.
3. Prepare two 10ml serological pipettes and a waste container. Set aside for later use.
4. Remove the dish from the incubator. Verify that the LifeSupport has melted to form a non-viscous liquid and that the Lifeink 240 collagen print has turned an opaque white color. If not, place it back into the incubator and monitor it until a change is observed. If so, proceed to the next step.
5. Pipette 10ml of LifeSupport out of the dish and place in the waste container. Be careful not to disturb the Lifeink 240 collagen bioprint with the pipette.
6. Pipette 10ml of 37ºC 1X PBS into the dish containing the LifeSupport and Lifeink 240 bioprint. Be careful not to disturb the Lifeink 240 collagen bioprint with the pipette.
7. Repeat steps 5 & 6 approximately 20 times, or until no gelatin remains in the dish or on the print.
8. If gelatin remains in the dish or on the print, place the dish back into the 37ºC incubator for 10 minutes, and then repeat steps 5 & 6 until no LifeSupport remains.
9. If using the AmScope 3WX2 LED-6WD LED Spot Light with Two 6500K LED Attachments: Place the 40 x 25mm glass dish on a stage with both LED lights suspended over it such that all the LED light will be concentrated within the dish. This should be approximately 75mm above the dish if using the same dish size. Turn the brightness control slider up to 10 (max). Do not turn on the LED light at this time.

If using another light source: adjust the setting of the light source to emit 425nm light at 53.3$mW/cm^{2}$

1. Pipette 0.6284 ml (628.4 µl) of the SPS solution into the 40 x 25mm dish. Discard the pipette tip.
2. Pipette 0.6284 ml (628.4 µl) of the Ru solution into the 40 x 25mm dish.
3. Use the pipette to gently draw liquid in and out, thereby mixing the solutions together in the dish. Complete this procedure approximately 20 times to ensure adequate mixing. Take caution not to disturb the collagen print.
4. Turn on the LED light. Expose the dish to the light for 15 minutes.
5. Use a spatula to remove the collagen bioprint from the dish. The crosslinking process is now completed.
6. Alternatively, you may also use a pipette to draw all of the solution out of the dish, which may make removing the print easier.
7. Store your completed bioprint suspended in 1X PBS or deionized water at 4ºC until ready for use.

**Disclaimer:** The opinions and assertions expressed herein are those of the authors and do not necessarily reflect the official policy or position of the Uniformed Services University (USU) or the Department of Defense. The opinions and assertions expressed herein are those of the authors and do not necessarily reflect the official policy or position of the Henry M. Jackson Foundation for the Advancement of Military Medicine (HJF), Inc.

**Acknowledgments:** This study was funded by the Center for Rehabilitation Sciences Research’s(CRSR) In-House Laboratory Independent Research (ILIR) award, Department of Physical Medicine and Rehabilitation, Uniformed Services University, Bethesda, MD, USA (award #HU00012320007)

**Authors’ contributions:** R.C.S., J.T.B, E.R.P., C.R.F, and S.H.J conceived the process and outlined the content. R.C.S. and J.T.B. contributed equally to writing the protocol and the accompanying article. X.Y. and S.H.J. provided guidance on the scope and content of the protocol. All authors reviewed, revised, and finalized the protocol.
